# *tmem33* is essential for VEGF-mediated endothelial calcium oscillations and angiogenesis

**DOI:** 10.1038/s41467-019-08590-7

**Published:** 2019-02-13

**Authors:** Aaron M. Savage, Sathishkumar Kurusamy, Yan Chen, Zhen Jiang, Karishma Chhabria, Ryan B. MacDonald, Hyejeong R. Kim, Heather L. Wilson, Fredericus J. M. van Eeden, Angel L. Armesilla, Timothy J. A. Chico, Robert N. Wilkinson

**Affiliations:** 1 Department of Infection, Immunity and Cardiovascular Disease, Medical School, University of Sheffield, Sheffield, UK; 2 The Bateson Centre, University of Sheffield, Sheffield, UK; 3 Cardiovascular Molecular Pharmacology Laboratory, Faculty of Science and Engineering, Research Institute in Healthcare Science, School of Pharmacy, University of Wolverhampton, Wolverhampton, UK; 4 Department of Biomedical Science, University of Sheffield, Sheffield, UK; 5 Sheffield CRISPRi Facility, Sheffield, UK

## Abstract

Angiogenesis requires co-ordination of multiple signalling inputs to regulate the behaviour of endothelial cells (ECs) as they form vascular networks. Vascular endothelial growth factor (VEGF) is essential for angiogenesis and induces downstream signalling pathways including increased cytosolic calcium levels. Here we show that transmembrane protein 33 (*tmem33*), which has no known function in multicellular organisms, is essential to mediate effects of VEGF in both zebrafish and human ECs. We find that *tmem33* localises to the endoplasmic reticulum in zebrafish ECs and is required for cytosolic calcium oscillations in response to Vegfa. *tmem33*-mediated endothelial calcium oscillations are critical for formation of endothelial tip cell filopodia and EC migration. Global or endothelial-cell-specific knockdown of *tmem33* impairs multiple downstream effects of VEGF including ERK phosphorylation, Notch signalling and embryonic vascular development. These studies reveal a hitherto unsuspected role for *tmem33* and calcium oscillations in the regulation of vascular development.

## Abstract (web summary)

Calcium signalling downstream of VEGF is essential for VEGF-induced angiogenesis. Here Savage et al. show that Transmembrane Protein 33 (TMEM33) is required for angiogenesis and the endothelial calcium response to VEGF, revealing a function for TMEM33 in multicellular organisms.

## Introduction

The formation of a complex vascular network is an essential process during embryonic development, which is vital for growth of tissues and is frequently dysregulated during disease in the adult. Endothelial cells (ECs) line the inner lumen of blood vessels and their organisation into complex branching networks requires co-ordination of molecular outputs coupled to specific cellular behaviours via a process primarily orchestrated by signalling from vascular endothelial growth factor (VEGF)^1^.

VEGF is a morphogen that signals via different ligands to induce motile and invasive behaviour, which drives blood vessel sprouting. VEGFA primarily controls angiogenesis from arteries via its cognate receptor VEGFR2/KDR, whereas VEGFC promotes sprouting from veins via VEGFR3/FLT4^2^. Migrating ECs extend filopodia to sense VEGF signals via *Kdr* (VEGFR2), as they form a new sprouting vessel^3^. Leading angiogenic ECs are termed tip cells, which upregulate *dll4* transcription, inducing Notch signalling in neighbouring cells, and this acts to limit excessive angiogenic sprouting^4^. Neighbouring Notch-expressing cells join the sprout as stalk cells, which in zebrafish tend to exhibit reduced proliferative capacity compared with tip cells^4,5^.

VEGFA has been shown to promote proliferation of ECs in vitro via VEGFR2-mediated activation of the RAS/RAF/ERK pathway without affecting migration^6^. Others, however, have shown that inhibition of ERK phosphorylation in vivo inhibits EC migration but not proliferation during angiogenesis^7^. ERK activation is induced via PLCG1 phosphorylation in vitro^8^, which generates inositol 1,4,5-trisphosphate (IP3). IP3 subsequently activates inositol triphosphate receptor (IP3R) Ca^2+^ channels within the endoplasmic reticulum (ER) to increase cytosolic Ca^2+^ concentrations and activate protein kinase C to phosphorylate ERK^9^. ERK activation is required to promote angiogenesis and has been shown to promote expression of tip cell markers including *dll4*^7,10^ and *flt4*^7^. VEGF and Notch therefore balance formation of tip and stalk cells within developing blood vessels and modulate the relative migration and proliferation of ECs during angiogenesis^11^. Ca^2+^ is a universal secondary messenger, which achieves specificity using complex signalling modalities. These include encoding information to activate cellular responses within cytosolic Ca^2+^ oscillations^12^. How EC Ca^2+^ oscillations integrate complex molecular outputs to precisely control discrete cellular behaviours in a developing vascular network remains unknown.

ECs are generally thought to be non-excitable and as such utilise store-operated calcium entry (SOCE) as their primary means to maintain Ca^2+^ levels in the ER after influx^13,14^. The principal trigger for SOCE activation occurs when intracellular calcium stores with the ER are depleted and these are refilled via interaction of TRPC1 and ORAI1 on the plasma membrane with ER-resident STIM1, which form the calcium-release activated channel^15–18^. SOCE relies on modulation of the actin cytoskeleton, which requires Ca^2+^-dependent proteins for ER motility^19^.

We have identified transmembrane protein 33 (TMEM33) as a component of the ER Ca^2+^ signalling machinery required for angiogenesis. TMEM33 is a three-pass transmembrane domain protein conserved throughout evolution with two paralogues in the budding yeast *Saccharomyces cerevisiae*, Pom33 and Per33, showing enrichment in the nuclear pore and ER, respectively^20,21^. In the fission yeast *Schizosaccharomyces pombe*, the TMEM33 orthologue Tts1p contributes to maintenance of the cortical ER network^22^. Human TMEM33 has been shown to localise to the nuclear envelope and ER in vitro, where it has been suggested to regulate the tubular structure of the ER by suppressing the membrane-shaping activity of reticulons^23,24^. However, its function within the ER in multicellular organisms remains unknown.

Here we describe the first characterisation of *tmem33* in a multicellular organism and show that *tmem33* is required in an EC-specific manner for Vegfa-mediated Ca^2+^ oscillations, to promote angiogenesis in zebrafish embryos. The requirement for *tmem33* during the response to VEGF is conserved from zebrafish to humans. Furthermore, *tmem33*-mediated endothelial Ca^2+^ oscillations are critical for formation of endothelial filopodia and contribute to activation of ERK and induction of Notch signalling to co-ordinate vascular morphogenesis.

## Results

### *tmem33* knockdown impairs vascular and pronephric development

**Fig. 1:**
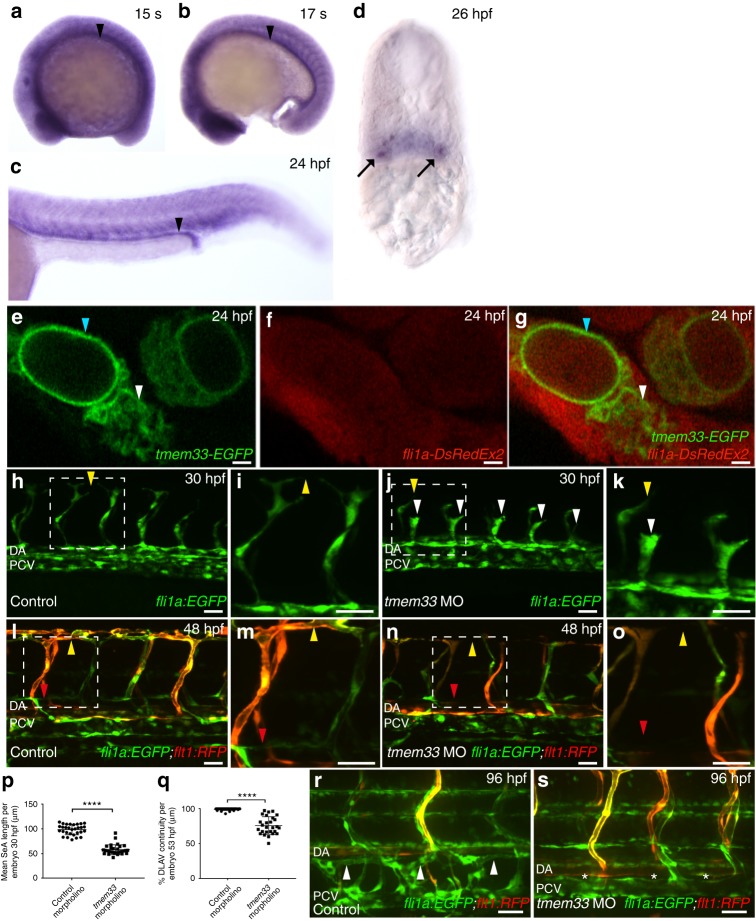
*tmem33* knockdown inhibits angiogenesis and localises to the ER in ECs. **a**–**d** *tmem33* is expressed ubiquitously during segmentation, but displays enrichment in the pronephros (black arrowheads) and somite boundaries, which is more pronounced from 24 hpf. Pronephric expression is evident in 26 hpf transverse sections (black arrows). **e**–**g** Tmem33-EGFP protein localises to the nuclear envelope (blue arrowheads) and ER (white arrowheads) within the caudal artery in *fli1a:DsRedEx2* embryos (Scale bars 1 µm). **h**–**k** *tmem33* morphants injected with 0.4 ng morpholinos display delayed migration of *Tg(fli1a:egfp)* positive SeAs, which stall at the horizontal myoseptum (**j**, **k**, white arrowheads), compared with control *Tg(fli1a:egfp)* positive morphants (**h**, **i**), which begin to anastomose by 30 hpf (yellow arrowheads) (scale bars 50 µm). **l**–**o** By 48 hpf, *Tg(fli1a:EGFP;−0.8flt1:RFP) tmem33* morphant SeAs complete dorsal migration, but display incomplete DLAV formation (**n**, **o**, yellow arrowheads) and lack lymphatic vasculature (red arrowheads). At 48 hpf *Tg(fli1a:EGFP; −0.8flt1:RFP)* control morphants display secondary angiogenesis (**l**, **m**, yellow arrowheads) and parachordal lymphangioblasts (red arrowhead) (scale bars 50 µm). **p** *tmem33* morphants injected with 0.4 ng morpholinos display reduced SeA length at 30 hpf (*t*-test ****p = < 0.0001; *t* = 4.075; DF = 24. *n* = 3 repeats, 10 embryos per group). **q** *tmem33* morphants injected with 0.4 ng morpholinos display incomplete formation of DLAV (*t*-test ****p = < 0.0001; *t* = 5.618; DF = 28. *n* = 3 repeats, 9 or 10 embryos per group). **r**, **s** Thoracic duct formation is impaired in *tmem33* morphants injected with 0.4 ng morpholinos (white asterisks), compared with control morphants (white arrowheads) (scale bars 50 µm). DA, dorsal aorta; PCV, posterior cardinal vein. Source data are provided as a Source Data file

We find *tmem33* is expressed ubiquitously during zebrafish segmentation (Fig. 1a–c) and by 26 h post fertilisation (hpf) is enriched in the trunk vasculature and pronephros (Fig. 1d). TMEM33 expression has been previously identified within the nuclear envelope and ER in human cells^23,24^. We expressed a full-length C-terminal *tmem33-EGFP* fusion messenger RNA in developing zebrafish embryos and found Tmem33-EGFP fusion protein to localise to structures indicative of nuclear envelope (Fig. 1e–g, blue arrowheads) and ER (Fig. 1e–g, white arrowheads) of ECs within the caudal artery.

To knock down *tmem33* expression and establish its function during embryonic development, we next used two splice blocking morpholinos targeting exon 3, (Supplementary Fig. 1a, b, Supplementary Tables 1 and 2). All experiments were conducted using co-injection of both morpholinos. *tmem33* morphants exhibited reduced segmental artery (SeA) length compared with controls (Fig. 1h-k, white arrowheads, p). By 30 hpf, SeAs in control morphants had reached the dorsal roof of the neural tube and begun to sprout laterally to form the dorsal longitudinal anastomotic vessel (DLAV) (Fig. 1h, i yellow arrowhead), whereas *tmem33* morphants often displayed abnormal spade-shaped tip cell morphology (Fig. 1k, white arrowhead) and were observed to stall at the level of the horizontal myoseptum. Consistent with this, *tmem33* morphants displayed reduced SeA migration rate (Supplementary Fig. 2a–d, yellow arrowheads, Supplementary Fig. 2e). Expression of an arterial marker *Tg(0.8flt1:RFP)*^25^ in *tmem33* morphants was indistinguishable from controls and restricted to the dorsal aorta (DA) and SeAs from day 1, suggesting the DA was correctly specified in *tmem33* morphants (Fig. 1l, n). Onset of circulation and blood flow was normal in *tmem33* morphants (Supplementary Fig. 3 and Supplementary Movies 1–3). Despite this early delay in sprouting angiogenesis, by 48 hpf, most SeAs in *tmem33* morphants had completed their dorsal migration, but many failed to migrate laterally, resulting in a primitive and discontinuous DLAV (Fig. 1l–o, yellow arrowheads, q). In addition, *tmem33* morphants displayed absent parachordal lymphangioblasts (Fig. 1l, n, red arrowheads). The thoracic duct (TD) is the first major lymphatic vessel to develop in zebrafish^26^ and forms by ventral migration of parachordal lymphangioblasts. In keeping with the absence of parachordal lymphangioblasts, the TD was absent in *tmem33* morphants (Fig. 1r, s, asterisks).

### *tmem33* knockdown reduces EC Ca^2+^ oscillations and filopodia

VEGF signalling is essential for SeA formation in zebrafish^7,27–31^ and lymphatic development^26,32–36^. Although TMEM33 has never been implicated in angiogenesis or VEGF signalling, the similarity of the phenotype of embryos with loss-of-function of *tmem33* or VEGF signalling led us to hypothesise that *tmem33* may regulate VEGF signalling in ECs. Furthermore, as VEGF increases EC intracellular Ca^2+^ ^37^, which is dependent upon the interaction of plasma membrane proteins with ER-resident proteins via SOCE^16,38^, we also postulated that *tmem33* may regulate VEGF-induced EC Ca^2+^ signalling, given its indicative expression in the ER (Fig. 1e–g).

To determine the effect of *tmem33* knockdown on EC calcium signalling in vivo, we generated an endothelial Ca^2+^ reporter line *Tg(fli1a:gal4FF*^*ubs3*^*;uas-gCaMP7a*^*sh392*^*)*, hereafter referred to as *fli1a:gal4FF*^*ubs3*^*;uas-GCaMP7a*. Recently, a similar transgenic has been employed as an indirect readout of VEGF signalling activity in zebrafish ECs^39^. To first establish whether *fli1a:gal4FF*^*ubs3*^*;uas-GCaMP7a* embryos reliably reported fluctuations in endothelial Ca^2+^ signalling, we treated embryos with Thapsigargin to inhibit the sarcoplasmic or ER Ca-ATPase (SERCA) family of Ca^2+^ pumps^40^ and thereby raise endothelial Ca^2+^ levels. Indeed, Thapsigargin treatment significantly increased GCaMP7a fluorescence in tip cells (Supplementary Fig. 4a,b,d) and reduced cytosolic Ca^2+^ oscillations in tip cells (Supplementary Fig. 4e). Conversely, as VEGF-mediated elevations in cytosolic Ca^2+^ are dependent upon IP3R function^37^, we treated embryos with an IP3R antagonist 2-APB, to reduce cytosolic Ca^2+^ (Supplementary Fig. 4a,c). *fli1a:GCaMP7a* embryos treated with 2-APB displayed reduced EC GCaMP7a fluorescence and reduced frequency of Ca^2+^ oscillations in tip cells (Supplementary Fig. 4a,c,d,e).

**Fig. 2:**
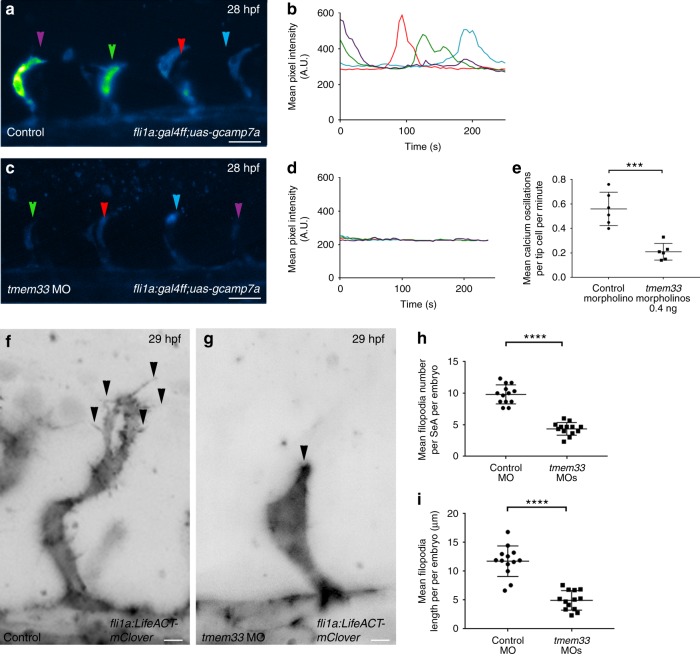
*tmem33* knockdown reduces Ca^2+^ oscillations in tip cells and reduces EC filopodia. **a**–**d** *tmem33* morphants injected with 0.4 ng morpholinos display reduced endothelial Ca^2+^ oscillations. Intensity projections show Ca^2+^ oscillations in both control and *tmem33* morphants, highlighting intensity over duration of the time lapse. Change in fluorescence over 300 s for four SeAs, trace colours correspond to equivalent arrowheads (scale bars 50 µm). **e** *tmem33* morphants display a significant reduction in Ca^2+^ oscillations when compared with control morphants (unpaired *t*-test ****p = < 0.001; *t* = 5.633; DF = 10, 2 repeats, *n* = 3 embryos per group). **f**, **g** *tmem33* morphants injected with 0.4 ng morpholinos display delayed migration of SeAs, which extend fewer filopodia, compared with controls (black arrowheads) (scale bars 5 µm). **h** *tmem33* morphants display significantly fewer filopodia per SeA compared with controls. Measurements taken from *n* = 4 SeAs per embryo. Unpaired *t*-test, ****p = < 0.0001; *t* = 7.805; DF = 24, 2 repeats, *n* = 6 or 7 embryos per group). **i** *tmem33* morphants display reduced filopodia length compared with controls, measurements taken from *n* = 4 SeAs. Unpaired *t*-test ****p < 0.0001; *t* = 10.86; DF = 24; 2 repeats, *n* = 6 or 7 embryos per group. Source data are provided as a Source Data file

Having established *fli1a:gal4FF*^*ubs3*^*;uas-GCaMP7a* as a reporter of EC cytosolic Ca^2+^ levels in vivo, we examined the effect of *tmem33* knockdown on EC Ca^2+^ oscillations*. tmem33* knockdown in *fli1a:gal4FF*^*ubs3*^*;uas-GCaMP7a* embryos significantly reduced the frequency of Ca^2+^ oscillations and GCaMP7a fluorescence intensity in EC tip cells (Fig. 2a–e and Supplementary Movies 4,5). Furthermore, treatment of *fli1a:gal4FF*^*ubs3*^*;uas-GCaMP7a* embryos with the VEGFR inhibitor Tivozanib/AV951 reduced Ca^2+^ oscillations in a similar manner to *tmem33* knockdown (Supplementary Fig. 5a–d,i). In contrast, *N*-[*N*-(3,5-Difluorophenacetyl)-L-alanyl]-S-phenylglycine t-butyl ester (DAPT)-mediated inhibition of Notch signalling, which negatively regulates VEGF signalling^41^, significantly increased cytosolic Ca^2+^ oscillations within EC tip cells (Supplementary Fig. 5e,f,i). These data indicate *tmem33* knockdown reduced endothelial Ca^2+^ activity to a similar level as VEGF inhibition and are consistent with studies that demonstrate EC calcium signalling mediates the response to VEGF in vivo^39^.

The delayed SeA sprouting, abnormal tip cell morphology and aberrant DLAV anastomosis following *tmem33* knockdown (Fig. 1h–o) are similar to phenotypes observed following inhibition of filopodia formation in endothelial tip cells^42^. Knockdown of *tmem33* in *Tg(fli1a:lifeACT-mClover)*^*sh467*^, which labels filamentous actin in ECs, substantially reduced the number and length of filopodia present on EC tip cells (Fig. 2f-i), suggesting defective filopodia formation may contribute to impaired angiogenesis in *tmem33* morphants. As filopodia are known to express VEGF receptors and transduce the migratory signal upon VEGF ligand binding^3,43^, we examined whether loss of filopodia could account for the reduction in EC Ca^2+^ oscillations observed in *tmem33* morphants (Fig. 2a–e, Supplementary Movies 4,5). *Tg(fli1a:lifeACT-mClover)* embryos treated with the actin depolymerising agent Latrunculin B did not form filopodia (Supplementary Fig. 6a–c, black arrowheads), instead forming rapidly depolymerising F-actin foci in keeping with previous reports^42^. Treatment of *fli1a:gal4FF*^*ubs3*^*;uas-GCaMP7a* embryos with Latrunculin B had no significant effect on frequency of endothelial Ca^2+^ oscillations in tip cells in comparison with controls (Supplementary Fig. 6d–f, white arrowheads). This suggests loss of EC filopodia in *tmem33* morphants is not responsible for reduced EC Ca^2+^ oscillations.

### *tmem33* mutant zebrafish embryos display genetic compensation

As morpholino knockdown is transient and may induce off-target effects, we next generated zebrafish *tmem33* mutants using transcription activator-like effector nucleases (TALENs)^44^. We identified a mutant allele (hereafter referred to as *sh443*) with a 2 bp deletion in exon 3, which induces a premature stop codon within exon 4 encoding the second conserved transmembrane helix (Supplementary Fig. 7a,b). *tmem33*^*sh443*^ mutants display substantially reduced expression of *tmem33* mRNA (Supplementary Fig. 7c–e) likely via nonsense-mediated decay, indicating this mutation represents a severe loss-of-function or null allele. However, in contrast to *tmem33* morphants, vascular (Supplementary Fig. 7f,g) and pronephric development was normal in *tmem33*^*sh443*^ mutants and homozygous *tmem33* mutant adults were viable. Recent studies describe mechanisms by which the zebrafish genome can compensate for genetic mutations but not morpholino knockdown^45^. Consistent with such compensation, homozygous *tmem33*^*sh443*^ mutants injected with *tmem33* morpholinos titrated to a level, which did not significantly induce *p53* expression (Supplementary Fig. 7h), displayed significantly longer SeAs compared with heterozygote or wild-type embryos (Supplementary Fig. 7i–l, arrowheads). In addition to the described effect on vascular and lymphatic development, *tmem33* morphants exhibited increased glomerular size (Supplementary Fig. 7m–o, arrows) and homozygous *tmem33*^*sh443*^ mutants were protected against the effect of the *tmem33* morpholino on glomerular size (Supplementary Fig. 7m–o, arrows). These data suggest *tmem33*^*sh443*^ mutants exhibit genetic compensation or transcriptional adaptation^46^.

### *tmem33* crispant embryos phenocopy *tmem33* morphant embryos

As *tmem33* mutants appeared to display genetic compensation, and in order to confirm the morpholino-induced phenotype, we next knocked down *tmem33* using Clustered Regularly Interspaced Short Palindromic Repeats interference (CRISPRi). This acts via steric inhibition of transcription^47^ and has been previously successful in zebrafish embryos^45^. In comparison with co-injection of mRNA encoding a catalytically inactive Cas9 nuclease (dCas9)^47^ and control single-guide RNAs (sgRNAs) lacking the dCas9 binding motif (Supplementary Table 2), co-injection of sgRNAs flanking the start codon of *tmem33* with dCas9 mRNA into zebrafish embryos (hereafter referred to as crispants) reduced *tmem33* expression by in situ hybridisation (Supplementary Fig. 8a,b, arrowhead) and quantitative reverse-transcriptase PCR (qRT-PCR) (Supplementary Fig. 8c). *tmem33* CRISPRi significantly reduced Ca^2+^ oscillations in tip cells (Supplementary Fig. 8d–h) and endothelial filopodia number and length (Supplementary Fig. 8i–l, arrowheads). We were able to rescue the angiogenic defects in *tmem33* morphants and crispants by co-injection of full-length *tmem33* mRNA (Supplementary Fig. 9) confirming that these were due to reduced expression of *tmem33*. Interestingly, at minimal phenotypic doses, induction of *p53* expression was substantially reduced in *tmem33* crispants in comparison with *tmem33* morphants injected with 4 ng morpholino (Supplementary Fig. 8m), suggesting CRISPRi may be less subject to nonspecific effects than morpholinos. Taken together, these data demonstrate that transcriptional inhibition of *tmem33* via CRISPRi induces endothelial defects highly similar to those generated by morpholino-mediated translational inhibition of *tmem33* (Fig. 2). These data indicate *tmem33* is required for endothelial cytosolic Ca^2+^ oscillations, endothelial filopodia formation and normal angiogenesis.

### *tmem33* promotes angiogenesis in an EC-specific manner

The morphant and crispant phenotypes described above were induced by global translational/transcriptional inhibition and *tmem33* is expressed in tissue other than ECs and the developing kidney. We therefore sought to examine the effect of EC- and pronephros-specific inhibition of *tmem33*. As CRISPRi employs a genetically encoded catalytically inactive Cas9 nuclease (dCas9)^47^, we postulated that co-expressing *dCas9* under the control of a tissue-specific promoter and gene targeting sgRNAs may facilitate tissue-specific knockdown of *tmem33*. We therefore generated *fli1a:dCas9*, *cryaa:cfp* and *enpep:dCas9*, *cryaa:cfp* constructs (Supplementary Fig. 10a,b) that drive transient dCas9 expression under control of either EC or pronephros-specific promoters, respectively. These constructs were injected alongside *Tol2* mRNA and gene-targeting sgRNAs to elicit tissue-specific gene knockdown.

**Fig. 3:**
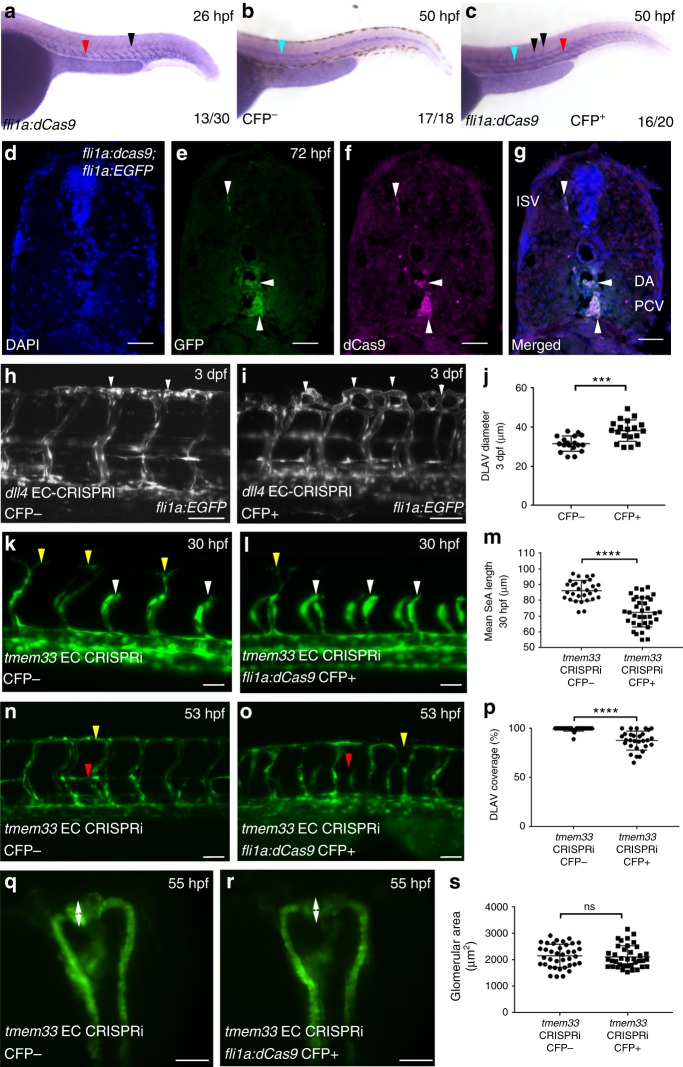
Injection of sgRNAs targeting *tmem33* into stable *Tg(fli1a:dCas9;cryaa:CFP)*^*sh512*^ embryos recapitulates knockdown of *tmem33*. **a** *dCas9* is expressed within the developing vasculature (red arrowhead) including SeAs (black arrowheads) in ~50% of progeny from a *Tg(fli1a:dCas9;cryaa:Cerulean)*^*sh512*^*/* + outcross at 26 hpf. At this stage the dominant *cryaa:Cerulean* marker is not expressed. **b** *dCas9* is not expressed within the developing vasculature in *CFP*^*-*^ *Tg(fli1a:dCas9;cryaa:Cerulean)*^*sh512*^ transgenic embryos at 50 hpf. Probe trapping in notochord is highlighted (blue arrowhead). **c** *dCas9* is expressed within the dorsal aorta (red arrowhead) and SeAs (black arrowheads) in *CFP*^*+*^ *Tg(fli1a:dCas9;cryaa:Cerulean)*^*sh512*^ transgenic embryos at 50 hpf. Probe trapping in notochord is highlighted (blue arrowhead). **d**–**g** Colocalisation of dCas9 and GFP in 72 hpf *fli1a:dCas9*; *fli1a:EGFP* embryo indicates EC restricted expression of dCas9 (white arrowheads) Scale bars 20 μm. **h**, **i** *Tg(fli1a:dCas9;cryaa:Cerulean)* embryos injected with sgRNA targeting *dll4* phenocopies ectopic vascular looping within the DLAV previously observed in *dll4* morphants and mutants at 3 dpf (white arrowheads). **j** *Tg(fli1a:dCas9;cryaa:Cerulean)*-positive embryos injected with sgRNAs targeting *dll4* display increased DLAV diameter (unpaired *t*-test ***p = < 0.001; *t* = 4.203 DF = 35; 2 repeats; *n* = 9 embryos per group). Scale bars 50 µm. **k-m** *Tg(fli1a:dCas9;cryaa:Cerulean)* embryos injected with sgRNAs targeting *tmem33* display reduced SeA length (yellow arrowheads highlight normal SeAs, white arrowheads highlight delayed SeAs) at 30 hpf (unpaired *t*-test ***p = < 0.0001; *t* = 6.716 DF = 62; 3 repeats; *n* = 9–12 embryos per group). Scale bars 50 µm. **n**–**p** *Tg(fli1a:dCas9;cryaa:Cerulean)* embryos injected with sgRNAs targeting *tmem33* display absent parachordal lymphangioblasts (red arrowhead) and reduced DLAV continuity (yellow arrowheads) (unpaired *t*-test ***p = < 0.0001; *t* = 6.399 DF = 56; 3 repeats; *n* = 9–11 embryos per group). Scale bars 50 µm. **q**–**s** *Tg(fli1a:dCas9;cryaa:Cerulean)* embryos injected with sgRNAs targeting *tmem33* display no significant difference in glomerular area, compared with *Tg(fli1a:dCas9;cryaa:Cerulean)*-negative siblings embryos (unpaired *t*-test, ns = not significant; *t* = 0.4048; DF = 74; 3 repeats; *n* = 12–13 embryos per group). Scale bars 200 µm. Source data are provided as a Source Data file

In comparison with co-injection of dCas9 mRNA and control sgRNAs lacking the dCas9-binding motif (Supplementary Fig. 10c, arrowheads, g arrows, k, l), co-injection of sgRNAs targeting *tmem33* and *dCas9* mRNA into progeny of an intercross between *Tg(fli1a:AC-TagRFP)*^*sh511*^*/*+ and *Tg(wt1b:egfp)/* + fish induced both abnormal vascular and pronephric development (Supplementary Fig. 10d, arrowheads h, arrows k, l,) as previously demonstrated. In contrast, embryos injected with *tmem33* sgRNAs and *fli1a:dCas9, cryaa:cfp* construct displayed significantly reduced DLAV continuity but normal kidney development (Supplementary Fig. 10e, arrowheads i, arrows k, l), whereas embryos injected with *tmem33* sgRNAs and *enpep:dCas9, cryaa:cfp* construct, restricting CRISPRi knockdown to the kidney, developed distended glomeruli, whereas the vasculature was normal (Supplementary Fig. 10f, arrowheads j, arrows k, l). We next generated a stable transgenic line expressing dCas9 in EC *Tg*(*fli1a:dCas9, cryaa-cerulean*)^*sh512*^, hereafter referred to as *Tg*(*fli1a:dCas9*). To avoid the potential for reduced of knockdown efficiency by fluorescent tagging, we employed untagged dCas9^45^. We screened for embryos expressing *dCas9* in ECs by in situ hybridisation (Fig. 3a–c) and validated EC-restricted expression of dCas9 using immunohistochemistry (Fig. 3d–g). As co-injection of dCas9 mRNA and control sgRNAs were consistent with normal vascular and pronephric development (Supplementary Fig. 8, S10), to test efficacy of EC gene knockdown in stable *dCas9* expressing transgenics, we first injected progeny of outcrosses from *Tg*(*fli1a*:*dCas9*)/+ × *Tg*(*fli1a:EGFP*)/+ with an sgRNA targeting *dll4* (Fig. 3h–j). Injection of sgRNA therefore served as an internal control in Cerulean Fluorescent Protein (CFP)-negative embryos. CFP-positive embryos displayed significantly increased DLAV diameter in comparison with CFP-negative embryos (Fig. 3h,i, arrowheads, j), consistent with previously observed ectopic DLAV sprouting in *dll4* morphants and mutants^48^. Injection of *tmem33* sgRNAs into *Tg*(*fli1a:dCas9*) embryos delayed SeA sprouting (Fig. 3k–m, compare white arrowheads with yellow arrowheads) and induced discontinuous DLAV formation (Fig. 3n–p, yellow arrowheads) only in CFP-positive embryos, similar to *tmem33* morphants (Fig. 1h–o) and crispants (Supplementary Fig. 10d,e, arrowheads, k). Furthermore, *Tg*(*fli1a:dCas9*) embryos injected with *tmem33* sgRNA displayed absent parachordal lymphangioblasts (Fig. 3n, o, red arrowheads) only in CFP-positive embryos, similar to *tmem33* morphants (Fig. 1l–o, red arrowheads). By contrast, *Tg*(*fli1a:dCas9*) embryos injected with *tmem33* sgRNA displayed no significant difference in glomerular area between CFP-positive and -negative embryos (Fig. 3q–s, arrows). Collectively, these data demonstrate tissue-specific CRISPRi-mediated uncoupling of *tmem33* functions within the endothelium and pronephros, and indicate EC-specific and pronephros-specific functions of *tmem33* during angiogenesis and pronephric development, respectively, in zebrafish. Importantly, all approaches to knockdown *tmem33* function were compatible with normal gross embryonic morphology at phenotypic doses (Supplementary Fig. 11).

### *tmem33* functions downstream of VEGF during angiogenesis

**Fig. 4:**
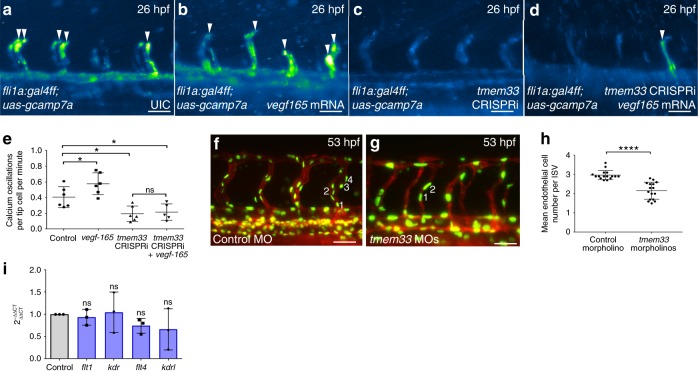
*tmem33* functions downstream of Vegfa during angiogenesis. **a**–**e** *tmem33* knockdown prevents Vegfa-mediated increases in Ca^2+^ oscillations. Overexpression of *vegfa*_*165*_ significantly increases frequency of Ca^2+^ oscillations in SeAs, whereas *tmem33* knockdown alone and in the presence of *vegfa*_*165*_ significantly reduce frequency of Ca^2+^ oscillations in SeAs. Intensity projection over time showing Ca^2+^ oscillations highlighting intensity change over duration of time lapse. Embryos display changes in fluorescence in SeA tip cells (white arrowheads) (two-way ANOVA with Holm–Sidak’s corrections: *p = < 0.05. 2 Repeats, *n* = 3 embryos per group) (scale bars 50 µm). **f**–**h** *tmem33* knockdown using 0.4 ng morpholinos reduces EC number in ISVs at 53 hpf. **h** Unpaired *t*-test; ****p < 0.0001; *t* = 6.507; df = 30; 2 repeats; *n* = 8 embryos per group. **i** VEGF receptor expression is not significantly affected in *tmem33* crispants (one-way ANOVA with Dunnett’s corrections; *p* = < 0.05, *n* = 3 repeats). Source data are provided as a Source Data file

Elevations in cytosolic Ca^2+^ are induced when EC tip cells respond to extracellular Vegfa via the Kdrl receptor^39^ and EC Ca^2+^ oscillations are reduced in tip cells following *tmem33* knockdown (Fig. 2a–e, Supplementary Movies 4, 5 and Supplementary Fig. 8d-h). We therefore sought to establish epistasis between VEGF signalling and *tmem33*. We overexpressed *vegfa*_*165*_ mRNA, which increases EC Ca^2+^ signalling (Supplementary Fig. 5g, h, j) and examined the effect on EC Ca^2+^ activity in the presence or absence of *tmem33* CRISPRi knockdown (Fig. 4a–e and Supplementary Movies 6–9). As expected, *vegfa*_*165*_ overexpression increased the frequency of cytosolic Ca^2+^ oscillations in endothelial tip cells (Fig. 4a, b, arrowheads, e) but this was significantly reduced by simultaneous *tmem33* knockdown by CRISPRi (Fig. 4d,e) similar to knockdown of *tmem33* alone without *vegfa*_*165*_ overexpression (Fig. 4c-e). Thus, *tmem33* is required for Vegfa-induced Ca^2+^ oscillations in endothelial tip cells.

In addition, Vegfa promotes proliferation of intersegmental vessel (ISV) ECs via a process normally limited by Notch signalling^4^. We therefore asked whether *tmem33* knockdown suppresses EC number in ISVs. Vessels are referred to as ISVs where the transgene employed did not allow distinction between SeAs and SeVs. Knockdown of *tmem33* reduced EC number within ISVs (Fig. 4f-h), similar to previous studies following VEGF inhibition^5^. Furthermore, VEGF receptor expression was unaffected in *tmem33* crispants (Fig. 4i). Taken together, these data demonstrate that *tmem33* mediates the effects of VEGF during angiogenesis and is required for VEGF-mediated cytosolic Ca^2+^ signalling within endothelial tip cells. Furthermore, these data suggest *tmem33* knockdown inhibits EC proliferation in response to VEGF.

### *TMEM33* is required for VEGFA-mediated angiogenesis in HUVECs

**Fig. 5:**
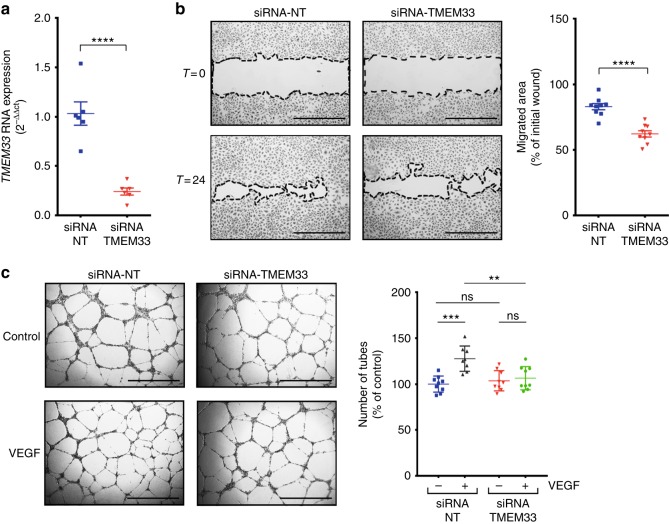
*TMEM33* is required for VEGFA-mediated angiogenesis in HUVECs. **a** *TMEM33* expression is significantly reduced by small interfering RNA (siRNA) knockdown. **b** Representative images of wound-healing migration assays performed with HUVECs transfected with a non-targeting control siRNA (siRNA-NT) or a siRNA specific for human TMEM33 (siRNA-TMEM33) after incubation in culture medium supplemented with VEGF (25 ng/ml). Data are shown as mean ± SE, *n* = 9, obtained from three independent experiments for comparison of cells transfected with siRNA-TMEM33 vs. siRNA-NT (unpaired two-tailed, Student’s *t*-test ****p ≤ 0.001). Images were taken at time zero and after incubation for 24 h. The migrated area was calculated by subtracting the value of the non-migrated area from the wound area at time zero and expressing this as a percentage of the total area at time zero. Scale bars, 1000 µm. **c** HUVECs transfected as described in **a** were plated on Growth-Factor Reduced Matrix (Geltrex) in Medium 200 containing 2% FBS (Control), supplemented with VEGF (25 ng/ml) when indicated (VEGF). Images show representative fields from experiments quantified in the histogram. Data are shown as mean ± SE, *n* = 8, obtained from three independent experiments; ns = nonsignificant; **p ≤ 0.01; ***one-way ANOVA with post-hoc Tukey’s comparison test *p* ≤ 0.005. Scale bars, 1000 µm. Source data are provided as a Source Data file

As *tmem33* mediates the effects of Vegfa in zebrafish, we next sought to establish whether this was conserved in humans. We used RNA interference to knock down *TMEM33* in human umbilical vein ECs (HUVECs) (Fig. 5a) and examined the effect on EC migration and tube formation in response to VEGFA (Fig. 5b). Consistent with our findings in zebrafish, *TMEM33*-deficient HUVECs migrated less than controls (Fig. 5b). In addition, HUVEC tubular morphogenesis induced by VEGFA was significantly reduced by *TMEM33* knockdown (Fig. 5c), indicating the requirement for *TMEM33* during the response to VEGFA is conserved in humans.

### *tmem33* knockdown reduces EC Notch and ERK signalling

**Fig. 6:**
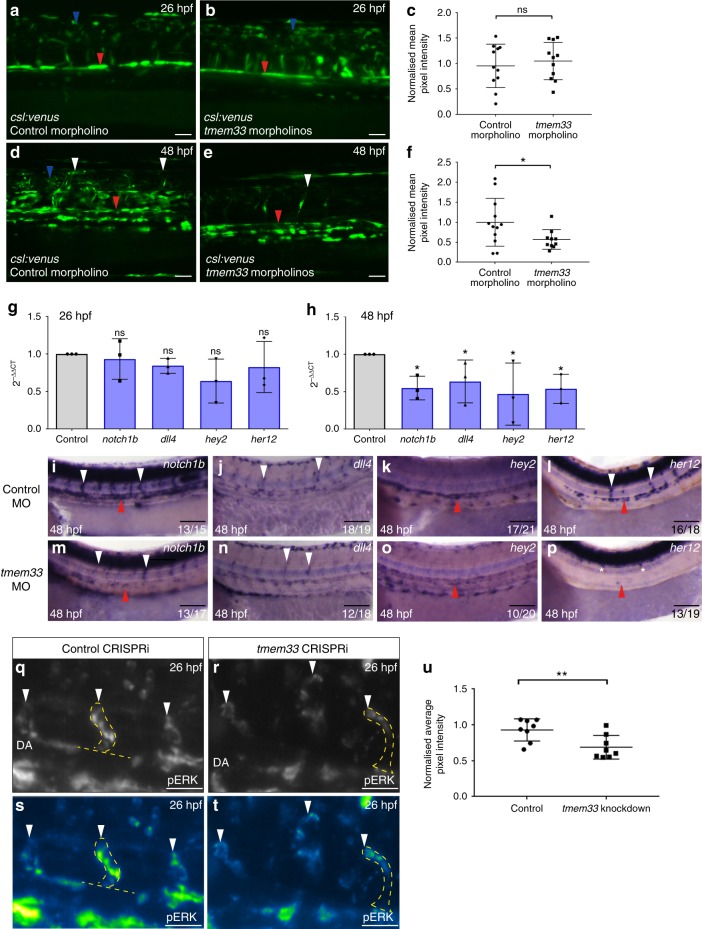
*tmem33* knockdown reduces endothelial Notch and ERK signalling during angiogenesis. **a**–**c** Notch reporter expression is unaffected by injection of 0.4 ng *tmem33* morpholinos at 26 hpf. Notch signalling activity in control embryos is present in neural tube (blue arrowhead) and DA (red arrowhead) (**c**, unpaired *t*-test; *t* = 0.5553; DF = 20; 2 repeats; *n* = 5 or 6 embryos per group). Scale bars 50 µm. **d**–**f** Injection of 0.4 ng *tmem33* morpholinos reduces Notch reporter expression at 48 hpf. Notch expression in control *Tg(csl:venus)* embryos is present in the neural tube (blue arrowhead), DA (red arrowhead) and SeAs (white arrowheads) (**f**, unpaired *t*-test; *p* = 0.0451; *t* = 2.138; DF = 20; 2 repeats; *n* = 6 and 4 embryos per group). Scale bars 50 µm. **g** Expression of *notch1b*, *dll4*, *hey2* and *her12* are not significantly altered by *tmem33* CRISPRi at 26 hpf (one-way ANOVA using post hoc Tukey’s comparison test. *n* = 3 repeats). **h** Expression of *notch1b, dll4, hey2* and *her12* are significantly reduced at 48 hpf by *tmem33* CRISPRi (one-way ANOVA using post hoc Tukey’s comparison test. *p = < 0.05; **p = < 0.01. *n* = 3 repeats). **i**–**p** In comparison with control morphants (**i**–**l**), embryos injected with 0.4 ng *tmem33* morpholino (**m**–**p**) display reduced expression of *notch1b* within the DA (**i**, **m**, red arrowheads) and SeAs (**i**, **m**, white arrowheads), *dll4* within SeAs (**j**, **n**, white arrowheads), *hey2* within the DA (**k**, **o**, red arrowheads) and *her12* within the DA (**l**, **p**, red arrowheads) and SeAs (**l**, **p**, white arrowheads). Scale bars 100 µm. **q**–**t** *tmem33* knockdown by CRISPRi reduces endothelial ERK phosphorylation (**q**, **r**, SeAs highlighted by yellow outline and white arrowheads). Intensity plot of ERK staining is shown (**s**, **t**, SeAs highlighted by yellow outline and white arrowheads). Scale bars 50 µm. **u** *tmem33* crispants display significantly reduced levels of pERK phosphorylation. Pixel intensity normalised to neural tube ERK fluorescence (unpaired *t*-test, **p = 0.0097, *t* = 2.993 DF = 14. *n* = 2 repeats, 4 embryos per group). Source data are provided as a Source Data file

VEGF signalling via VEGF receptor 2 (*kdr*), and VEGFR4 (*kdrl*) in zebrafish, induces transcription of *dll4* in endothelial tip cells via the MEK-ERK signalling pathway. Upregulation of Dll4 in endothelial tip cells induces Notch signalling in neighbouring stalk cells, which balance tip and stalk cell identity via regulation of VEGFR expression^7,41^. As *tmem33* mediates the response to VEGF, we examined whether Dll4/Notch signalling was perturbed by *tmem33* knockdown. Using a transgenic Notch reporter *Tg(csl:venus)*^49^, which reliably reports endothelial Notch signalling (Supplementary Fig. 12a–f), we observed no significant differences in reporter expression at 26 hpf (Fig. 6a, b red arrowhead, c), but by 48 hpf, Notch reporter expression was significantly reduced in the DA (Fig. 6d, e, red arrowheads, f) and SeAs displayed reduced Venus fluorescence in *tmem33* morphants (Fig. 6d, e, white arrowheads). In keeping with this, expression of *dll4*, *notch1b* and the Notch targets *hey2/gridlock* and *her12* were reduced by *tmem33* knockdown using global CRISPRi at 48 hpf but not 26 hpf (Fig. 6g, h and Supplementary Table 3). In situ hybridisation confirmed reduced arterial expression of all genes assayed by qPCR at 48 hpf (Fig. 6i–p, arrowheads). Phosphorylation of the serine/threonine kinase ERK preferentially occurs in angiogenic ECs^7^. In keeping with this requirement downstream of VEGF signalling during angiogenesis, *tmem33* knockdown reduced phosphorylated ERK (pERK) in sprouting SeAs (Fig. 6q–t, white arrowheads, u). pERK promotes expression of *dll4* in zebrafish angiogenic ECs^7,10^ and *dll4* expression was reduced by *tmem33* knockdown (Figs. 6h, n, arrowheads). These data indicate *tmem33* is required for ERK phosphorylation and Notch signalling, which act downstream of VEGF in angiogenic ECs.

As *tmem33* promotes sprouting angiogenesis cell autonomously (Fig. 3), we examined whether several other molecular and cellular consequences of *tmem33* inhibition were due to *tmem33* function within ECs. Similar to knockdown of *tmem33* by morpholino and CRISPRi, *Tg*(*fli1a:dCas9*) embryos injected with *tmem33* sgRNA displayed reduced endothelial Ca^2+^ oscillations (Supplementary Fig. 13a–c, white arrowheads), reduced filopodia number and length (Supplementary Fig. 13d–g, black arrowheads), reduced EC number in ISVs (Supplementary Fig. 13h–j) and reduced endothelial Notch signalling (Supplementary Fig. 13k–m, arrowheads) in CFP-positive embryos in comparison with CFP-negative siblings. Collectively, this indicates *tmem33* is required cell autonomously to promote endothelial Ca^2+^ oscillations, filopodia formation, Notch signalling and to regulate EC number during angiogenesis.

### SOCE inhibition inhibits angiogenesis

**Fig. 7:**
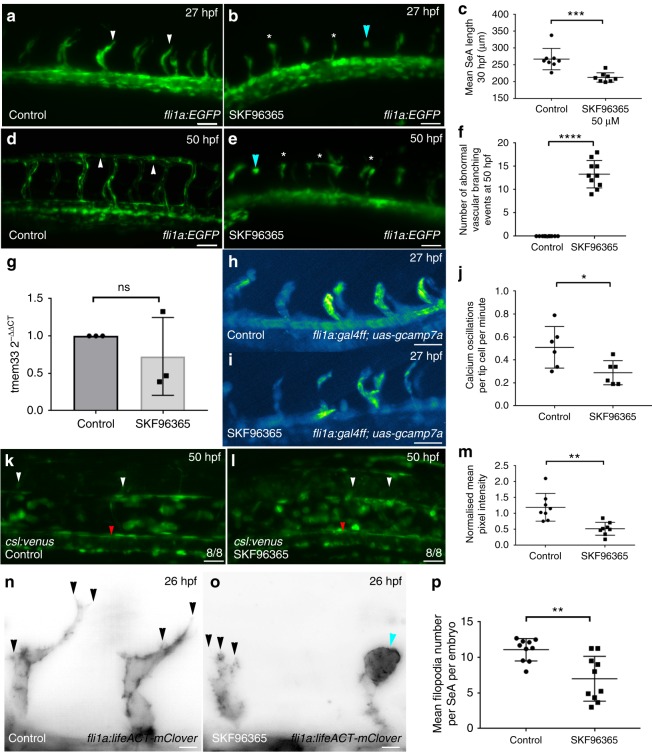
SOCE inhibition limits sprouting angiogenesis and disrupts signalling pathways downstream of VEGF. **a**, **b** SKF9635 impairs angiogenesis in *Tg(fli1a:EGFP)* embryos (white arrowheads) and induces abnormal tip cell morphology (blue arrowhead) by 27 hpf. Scale bars 50 µm. **c** SKF-96365-treated embryos display reduced SeA length by 27 hpf (unpaired *t*-test; *p* = < 0.0001; *t* = 11.22 DF = 14; 2 repeats, *n* = 4 embryos per group). **d**, **e** SKF-96365 treatment impairs DLAV formation (white asterisks) in comparison with control embryos, which form the DLAV normally by 50 hpf (white arrowheads). Abnormal tip cell morphology (blue arrowhead) remain in SKF-96365-treated embryos by 50 hpf. Scale bars 50 µm. **f** SKF-96365-treated embryos display increased frequency of abnormal vascular branching (stunted SeAs or incomplete DLAV anastomosis) by 50 hpf (unpaired *t*-test; *p* = < 0.0001; *t* = 14.28 DF = 18. *n* = 2 repeats, *n* = 5 embryos per group). **g** *tmem33* expression is not significantly altered by SKF-96365 treatment (unpaired *t*-test; *t* = 0.911, DF = 4, *n* = 3 repeats). **h**–**j** SKF-96365 reduces frequency of endothelial Ca^2+^ oscillations in *Tg(fli1a:gal4FF*^*ubs3*^*;uas-gcamp7a)* embryos. Intensity projection over time showing calcium oscillations in control embryos, highlighting intensity change over duration of time lapse (unpaired *t*-test; *p* = < 0.05; *t* = 2.594, DF = 10, *n* = 2 repeats, 3 embryos per group). **k**, **l** Notch reporter expression is reduced in SeAs (white arrowheads) and DA (red arrowhead) of *Tg(csl:venus)* embryos. Scale bars 50 µm. **m** DA expression of Notch reporter is reduced by SKF treatment (unpaired *t*-test, **p = 0.0097, *t* = 3.982 DF = 15, 2 repeats, *n* = 4 embryos per group). **n**, **o** SKF-96365 treatment reduces endothelial filopodia number (black arrowheads) and abnormal spade-shaped tip cell morphology (blue arrowhead), in *Tg(fli1a:lifeact-mClover)* embryos. Scale bars 5 µm. **p** SKF-96365 treatment reduces mean filopodia number per SeA per embryo by 26 hpf (unpaired *t*-test; *p* = < 0.05; *t* = 2.525, DF = 10, *n* = 2 repeats, *n* = 5 embryos per group). Source data are provided as a Source Data file

**Fig. 8:**
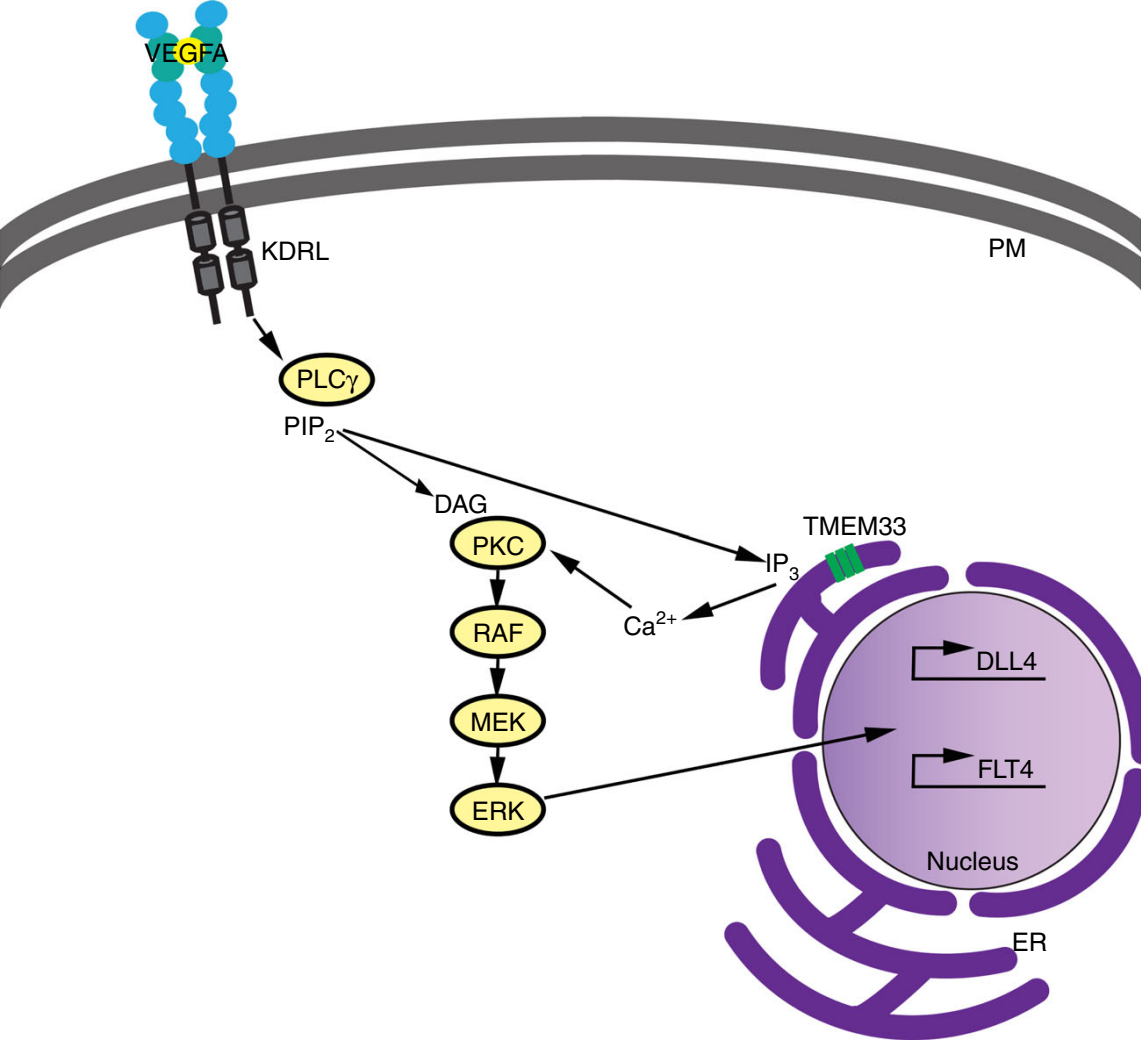
Proposed model for *tmem33* function in endothelial cells during angiogenesis. When VEGFA binds to its cognate receptor, e.g., Kdrl, the resulting phosphorylation of PLCγ generates inositol 1,4,5-trisphosphate (IP3) and this, in turn, binds to IP3 receptors on the ER membrane, which release Ca^2+^ into the cytosol from intracellular stores. Our data suggest the VEGF-mediated release of Ca^2+^ from ER stores during angiogenesis is dependent on Tmem33 function within the ER membrane. Furthermore, resultant Ca^2+^ oscillations generated in tip cells downstream of Vegfa contribute to phosphorylation of ERK and induction or maintenance of downstream targets including Dll4/Notch signalling, to co-ordinate cellular behaviours during vascular morphogenesis. ER, endoplasmic reticulum; P, plasma membrane

Although *tmem33* knockdown impairs angiogenesis and reduces endothelial cytosolic Ca^2+^ oscillations, it was possible that these effects were due to different roles of *tmem33* rather than causally linked. We therefore sought to determine whether inhibiting EC cytosolic Ca^2+^ release was sufficient to cause the observed phenotype of *tmem33* knockdown. We inhibited SOCE using SKF-96365, a compound that inhibits STIM1 and TRPC1 function^7,50^. SKF-96365 treatment between 21 hpf and 27 hpf reduced SeA length (Fig. 7a–c arrowheads) as induced by *tmem33* knockdown (Fig. 1j, k white arrowheads). Embryos treated from 21 hpf until 50 hpf displayed incomplete DLAV formation (Fig. 7d–f, arrowheads) similar to *tmem33* knockdown (Fig. 1n, o). Importantly, although *tmem33* expression was unaffected by SKF-96365 treatment (Fig. 7g), frequency of endothelial Ca^2+^ oscillations was significantly reduced in tip cells (Fig. 7h–j). Furthermore, Notch reporter expression (Fig. 7k–m) and endothelial tip cell filopodia number (Fig. 7n–p) was reduced in ECs following SKF-96365 treatment similar to *tmem33* knockdown (Fig. 6d–f and Fig. 2f-i, respectively). SKF-96365-treated embryos also displayed abnormal spade-shaped tip cell morphology (Fig. 7b, e, o, blue arrowheads) as induced by *tmem33* knockdown (Fig. 1k, white arrowhead). Given the similar angiogenic defects induced by *tmem33* knockdown and SKF-96365 treatment, we examined whether inhibition of SOCE could account for reduced EC number and migration previously observed in *tmem33* morphants (Fig. 4f–h, and Supplementary Fig. 2). We treated embryos from 21 hpf until 30 hpf and quantified EC proliferation and migration in the presence and absence of SKF-96365 between 24 and 30 hpf (Supplementary Fig. 14 and Supplementary Movies 10, 11), and observed that both SeA migration (Supplementary Fig. 14a–e, arrowheads) and tip and stalk cell proliferation (Supplementary Fig. 14a–d,f–h) were significantly reduced in migrating SeAs treated with SKF-96365. Consistent with this, expression of VEGF receptors in *tmem33* crispants were unaffected (Fig. 4i) and thus unlikely to account for reduced EC number (Fig. 4f–h and Supplementary Fig. 13h–j). Collectively, these data demonstrate that SOCE inhibition impairs angiogenesis in vivo by limiting EC migration, proliferation, disrupting signalling pathways downstream of VEGF and inhibiting filopodia formation similar to *tmem33* knockdown. This is consistent with our hypothesis that reduced cytosolic EC Ca^2+^ oscillations account for the angiogenic defects observed caused by *tmem33* knockdown. Thus, *tmem33* is essential for VEGF-mediated endothelial Ca^2+^ oscillations that are required for tip cell filopodia formation, the downstream consequences of VEGF signalling and developmental angiogenesis (Fig. 8).

## Discussion

No previous study has examined the function of *tmem33* in a multicellular organism. Previous studies in yeast^20–22^ and transformed human cells^23,24^ had localised *tmem33* to the ER, but no function has been demonstrated during development. We find *tmem33* is expressed widely throughout the developing zebrafish embryo and localised to structures indicative of ER in zebrafish ECs. In keeping with its expression patterns, *tmem33* knockdown demonstrated a requirement for *tmem33* during normal angiogenesis and pronephric development. Furthermore, TMEM33 is also required for VEGFA-mediated angiogenesis in human ECs, indicating a conserved function between zebrafish and human.

We find that *tmem33* is required for Vegfa-mediated cytosolic Ca^2+^ oscillations in endothelial tip cells. Although increases in cytosolic Ca^2+^ are a well-established response to VEGF in ECs^37^, it remained unclear whether Ca^2+^ signalling was required for its effects in vivo and, if so, how Ca^2+^ co-ordinated EC behaviours during angiogenesis. Recent studies have established that Vegfa/Kdrl signalling is required for endothelial Ca^2+^ oscillations during sprouting angiogenesis in vivo^39^. Our findings are consistent with these, as VEGFR inhibition abrogated Ca^2+^ oscillations and overexpression of *vegfa*_*165*_ mRNA or Notch inhibition increased Ca^2+^ oscillations in tip cells. Reduction of Ca^2+^ oscillations in tip cells following either *tmem33* knockdown or acute SOCE inhibition were associated with reduced formation of filopodia, abnormal tip cell migration and aberrant anastomosis. Impaired SeA migration and DLAV anastomosis have been observed in embryos with reduced EC filopodia formation, suggesting this may contribute to the angiogenic defects induced by *tmem33* knockdown^42^. Importantly, the frequency of cytosolic Ca^2+^ oscillations in tip cells was unaffected following loss of filopodia. This is in keeping with studies that demonstrated tip cells without filopodia retained their ability to respond to Vegfa^42^ and suggests reduced filopodia formation induced by *tmem33* knockdown was secondary to reduced EC Ca^2+^ oscillations. Ca^2+^ oscillations have been reported to promote F-actin reorganisation and cell migration in vitro^51,52^. It therefore seems likely to be that *tmem33*-mediated cytosolic Ca^2+^ oscillations induced by VEGF signalling promote tip cell migration in vivo via organisation of the actin cytoskeleton. As such, *tmem33*-mediated Ca^2+^ oscillations permit tip cells to respond quickly to the proangiogenic Vegfa stimulus.

Vegfa-mediated activation of ERK signalling is also required for angiogenic sprouting and induction of tip cell marker expression including *dll4* ^7,10,33,53^. *tmem33* knockdown reduced ERK phosphorylation and Dll4/Notch signalling, and delayed tip cell migration in a similar way to ERK inhibition^7,53^ without significantly affecting *vegfr* expression. This indicates *tmem33* function contributes to activation of these pathways in vivo, likely to be via Ca^2+^-dependent mechanisms downstream of Vegfa. Interestingly, induction of Dll4/Notch signalling at 26 hpf was not dependent on *tmem33* function; however, its attenuation at 48 hpf, suggests *tmem33* contributes to maintenance of Dll4/Notch signalling in ECs. Consistent with this, inhibition of SOCE reduced Notch reporter activity at 48 hpf, suggesting an input from VEGF-mediated calcium signalling between 1 and 2 dpf to maintain Dll4/Notch signalling within developing arteries. Furthermore, recent studies indicate a requirement for Ca^2+^ binding during folding of Notch receptors and ligand engagement, including the Dll4–Notch1 interaction^54^. Reduced expression of *dll4* induced by *tmem33* knockdown is in keeping with observed reductions in ERK phosphorylation. ERK phosphorylation is required for development of the lymphatic system^33-35,55^; therefore, reduced ERK phosphorylation induced by *tmem33* knockdown may contribute to defective lymphatic sprouting.

Surprisingly, *tmem33* mutants exhibited normal angiogenesis, yet displayed substantial reductions in *tmem33* expression. The relative ease with which one can now generate targeted lesions within the zebrafish genome and widespread adoption of these approaches by the community has generated substantial evidence that many loss-of-function mutants fail to recapitulate morpholino-induced phenotypes^56^. Although this was initially attributed to well-established off-target effects of morpholinos^57^, recent studies have indicated a greater degree of compensation within the zebrafish genome than previously considered^45^. The *tmem33*^*sh443*^ allele is predicted to generate a premature stop codon within coding exon 4, located 53 nucleotides downstream of an exon junction. This is consistent with the requirement for induction of nonsense-mediated decay^58^ and observed reductions in *tmem33* expression. It is therefore likely that this allele represents a severe loss of function or null mutation, and that compensatory machinery exists within the zebrafish genome to account for normal angiogenesis in *tmem33*^*sh443*^ mutant embryos.

To circumvent these compensatory pathways, we have developed a strategy to transiently knock down gene function within zebrafish endothelium and pronephros using tissue-specific CRISPRi. Using this approach we were able to uncouple tissue-specific functions of *tmem33* and show that *tmem33* is required cell autonomously in both tissues for their normal development. *tmem33* functions within ECs to promote endothelial Ca^2+^ oscillations and filopodia formation during angiogenesis, and also to promote Notch signalling and regulate EC number. Tissue-specific CRISPRi offers a relatively straightforward method to study gene function within different tissues and represents an alternative to laborious cell transplantation procedures. Tissue-specific CRISPRi can be employed transiently to uncouple tissue-specific functions of genes, via co-injection of gene-targeting sgRNAs, dCas9 expression constructs and *Tol2* mRNA. However, we observed more severe defects using this approach than when using stable transgenic lines expressing dCas9. We suspect this may be due to variable copy number integration mediated by Tol2 and thus variable dCas9 expression, in addition to inherent toxicity of DNA microinjections. At minimal phenotypic doses, CRISPRi is less prone to induction of *p53* than some morpholinos and although this will vary depending upon the sgRNA, target and morpholino employed, our experience with the genes we have studied to date suggests CRISPRi may be susceptible to fewer off-target effects.

The localisation of *tmem33* within the ER in zebrafish ECs suggest it may have an important function in regulation of endothelial SOCE. Consistent with this, acute antagonism of SOCE induced similar reductions in cytosolic Ca^2+^ oscillations, filopodia formation and endothelial Notch activity as *tmem33* knockdown, without affecting *tmem33* expression. Furthermore, SOCE inhibition reduced EC proliferation, consistent with reduced EC number induced by *tmem33* knockdown. Genome-wide protein interaction studies in *Drosophila* embryos, have shown that the *Drosophila* Tmem33 orthologue, Kr-h2, physically interacts with SERCA^59^, and although this requires functional validation, the interaction appears to be conserved in humans. This suggests an ancestral function of Tmem33 in regulation of SOCE. Tmem33 may also act to augment the activity of Ca^2+^ channels such as IP3R within the ER. Alternatively, *tmem33* may be involved in mediating cortical distribution of the ER, as has been described for a yeast orthologue Tts1p^22^. Interestingly, rapid changes in cytosolic Ca^2+^ are known to induce dynamic changes in actin cytoskeleton organisation in vitro^60^. The actin cytoskeleton has been linked to normal SOCE function by facilitating interaction of ER-resident proteins with their plasma membrane counterparts^19,61^. As *tmem33* knockdown reduces Ca^2+^ oscillations and inhibits filopodia formation, this represents an intriguing possibility. VEGF has been shown to induce distinct modalities of calcium oscillations that correlate with different EC behaviours, including migration and proliferation^62^. We find that inhibition of SOCE impairs both migration and proliferation of ECs and knockdown of *tmem33* similarly impairs EC migration and reduces EC number, suggesting an essential requirement for Ca^2+^oscillations in both processes. The endothelial actin cytoskeleton is necessary for efficient mitosis and migration, and is notably disrupted by *tmem33* knockdown or SOCE inhibition. This suggests that reduced EC proliferation and migration induced by inhibition of SOCE or reduced migration and EC number induced by *tmem33* knockdown may represent a consequence of Ca^2+^-dependent disruption of the endothelial actin cytoskeleton. Further studies will be required to address the precise function of Tmem33-mediated Ca^2+^ within the ER in ECs in response to VEGF.

## Methods

### Zebrafish strains, morpholinos and sgRNA

All zebrafish were maintained according to institutional and national ethical and animal welfare guidelines. All experiments were performed under UK Home Office licences 40/3708 and 70/8588. The following published zebrafish lines were employed: *Tg(fli1a:EGFP)*^*y1*^ ^63^ *Tg(−0.8flt1:enhRFP)*^*hu5333*^ ^25^, *Tg(−26wt1b:EGFP)*^*li1*^ ^64^, *Tg(flk1:EGFP-NLS)*^*zf109*^ ^65^, *Tg(kdrl:HRAS-mCherry-CAAX)*^*s916*^ ^66^ and *Tg(csl:venus)*^*qmc61*^^49^. For details on mutant and transgenic line generation, see Supplementary Methods. A quantity of 0.2 or 2 ng of Sp2b and Sp3 morpholinos were injected. Where these were co-injected, a total of 0.4 ng or 4 ng was injected. Four nanograms of morpholino was found to exhibit some induction of p53 (Supplementary Fig. 7h) and was titrated to 0.4 ng to reduce this; however, phenotypes were equivalent at each dose. One nanogram of each sgRNA was injected alongside 500 pg of *dCas9* mRNA^45^. Negative control sgRNAs contained the DNA-binding element of the active gRNA but lacked the stem-loop forming region associated with binding to the Cas9 or dCas9 protein. For morpholino, sgRNA and primer sequences, see Supplementary Methods.

### Generation of *tmem33*^*sh443*^ mutant allele

TALENs specific for *tmem33* (ENSDARG00000041332) were designed against the following sequence 5′-cttcctggcccaggctt-3′ targeting an EcoNI site within exon 3. TALENs were assembled using the Golden Gate TALEN and TAL Effector Kit (Addgene, MA, USA)^44^ to generate the pTmem33Tal1L&R plasmids. Following linearisation of pTmem33Tal1L&R with NotI, capped mRNA was generated by in vitro transcription and 1500 pg TALEN mRNA was injected per embryo. Individual G0 embryos were tested by PCR and restriction fragment length polymorphism using tmem33ex3F and tmem33ex3R primers (Table S1), to identify somatic mutations that destroyed the EcoNI restriction site. The progeny of TALEN-injected G0 adults were incrossed and genotyped to confirm the presence of the *sh443* allele. All studies were performed using F3 and F4 generations.

### Generation of *fli1a:dCas9* and *enpep:dCas9* constructs

pME-dCas9 was generated by cloning the *nls-dCas9-nls* coding sequence from pT3TS-dCas9, a kind gift of Didier Stainier, into pME-MCS2, which contained the multiple cloning site of pCS2+. *fli1a:dCas9;cryaa:cfp* and *enpep:dCas9;cryaa:cfp* constructs (Supplementary Fig. 10a) were subsequently generated using the *Tol2* Kit via standard methods^67^. Each construct was injected into Nacre embryos alongside *Tol2* mRNA at 25 pg/nl.

### Generation of transgenic lines

Tg(*fli1a:Gal4FF*^*ubs3*^;*UAS-GCaMP7a*)^*sh392*^ was generated via injection of pTol2UASGCaMP7a into progeny of *tg*(*fli1a:Gal4FF*)^ubs3^ ^68^ heterozygous outcross alongside *tol2* mRNA according to standard protocols^67^. *Tg(fli1a:LifeAct-mClover)*^*sh467*^ was generated by fusing the LifeAct F-Actin binding motif^69^ to the N terminus of mClover by PCR (Table S1) to generate pME-LifeAct-mClover. *fli1a:LifeAct-mClover* construct was generated using the *tol2* Kit via standard methods^67^ and the following components: *fli1a* enhancer/promoter^70^, pME-LifeAct-mClover, pDestTol2-pA2^67^, and p3E-SV40pA^67^. Tg(*fli1a:DsRedEx2*)^*sh495*^ was generated using the *tol2* Kit via standard methods^67^ and the following components: *fli1a* enhancer/promoter^70^, pENTRDSRedEx2^70^, pDestTol2-pA2^67^, and p3E-SV40pA^67^. Tg(*fli1a:AC-TagRFP*)^*sh511*^ was generated by amplifying the actin-V_H_H-TagRFP coding sequence from pAC-TagRFP (Chromotek) and adding attB1/B2R sites (Table S1) to generate pME-AC-TagRFP. *fli1a:AC-tagRFP* construct was generated using the *Tol2* Kit^67^ and components listed above. *Tg(fli1a:dCas9, cryaa:Cerulean)*^*sh512*^ was generated by co-injecting *fli1a:dCas9, cryaa:cfp* plasmid with *Tol2* mRNA. Embryos were injected at one-cell stage with 25 ng/μl *Tol2* mRNA and corresponding plasmid DNA.

### mRNA microinjections

Microinjections of both *vegfa*_*165*_^63^ (300 pg), *tmem33* (250 pg) and *tmem33-gfp* (1200 pg) mRNA were performed on single-cell-stage embryos with 1 nl injection volume.

### RNA in situ hybridisation and immunohistochemistry

Alkaline phosphatase wholemount in situ hybridisation experiments were performed using standard methods as described previously^71^. Detailed protocols are available upon request. EC *dCas9* expression was detected using Cas9 in situ probe^72^. Immunohistochemistry to detect pERK was performed using Phospho-p44/42 Erk1.2 (Thr202.Tyr204) Rabbit mAb (#4370, cell signal, 1:250) and quantified by normalising EC signal against ERK staining within the neural tube as described^7,73^. Immunohistochemistry to detect dCas9 was performed on whole-mount and sectioned embryos as described^74^ using mouse anti-CRISPR/Cas9 (7A9-3A3) (Novus Biologicals, NBP2-36440, 1:100), chicken anti-GFP (Abcam, ab13970, 1:500) primary antibodies, goat anti-mouse IgG (H&L) Alexa Fluor® 647 (Thermo Fisher A21235, 1:1000), and goat anti-chicken IgY (H&L) Alexa Fluor® 488 (Thermo Fisher A11039, 1:1000) secondary antibodies. Sectioned embryos were fixed at 3 dpf in 4% paraformaldehyde overnight, washed into 30% sucrose and sectioned at 14 μm thickness on a Jung Frigocut cryostat (Leica).

### Quantitative RT-PCR

qRT-PCR was performed using Taqman™ assays according to the manufacturer’s instructions (see Supplementary Methods for probe details). RNA was extracted from batches of 20 or 30 whole embryos per repeat using Trizol®, as a template for complementary DNA synthesis (Verso™ cDNA synthesis kit, Thermo Fisher). A 7900 Real-Time PCR system (Applied Biosystems) was used for qPCR experiments. Gene expression levels were normalised to *eef1α* and for each comparison the experimental group was normalised to their relative control unless otherwise stated. All experiments were performed in triplicate. Results display triplicate 2^−ΔΔCT^ values and SEM (unless otherwise stated).

### Image acquisition and analysis

For light sheet imaging, embryos were anaesthetised using tricaine in E3 medium and mounted in 1% agarose. A light sheet Z.1 system was used and images were acquired using ZEN software (Zeiss). For confocal microscopy, still images were taken using an Ultraview VOX confocal spinning disc system (Perkin Elmer), Zeiss LSM880 with Airyscan and Leica SP5. Images were analysed using Volocity®V5.3.2, ZEN software (Zeiss) and Leica LCS Confocal software. In cases where samples displayed drift during time lapses, these were corrected using the translation algorithm in ZEN (Blue Edition).

### Calcium imaging

Calcium oscillations were quantified using 12–14 SeAs between 24 and 28 hpf on a Zeiss Lightsheet Z.1. All time lapses were taken at 3 µm *z*-intervals at 20 frames per second (fps) with an acquisition time of 4–6 s per stack using a × 20 objective lens for 300 s. Changes in fluorescence over time were measured throughout the DA to establish a baseline. Changes in fluorescence measured in tip cells were determined to be peaks by subtracting mean DA fluorescence. Changes in fluorescence were normalised to DA baseline values. In experiments where ectopic Ca^2+^ oscillations were induced in the DA, the mean DA value of control embryos was used to normalise datasets. Absolute calcium peak number was quantified and averaged per SeA, per minute. FIJI was employed to quantify fluorescence intensity. Tip cells were manually segmented as regions of interest and mean and peak fluorescence (arbitrary units) were determined.

### Pharmacological treatment

VEGF inhibition using Tivozanib/AV951 (500 nM), Notch inhibition using DAPT (100 μM) or inhibition of F-actin polymerisation using Latrunculin B (380 nM) was conducted using dechorionated embryos between 24 and 28 hpf in E3 medium with dimethyl sulfoxide (DMSO) as a control. SERCA inhibition using Thapsigargin (5 μM) was performed for 45 min before immediate imaging using a Perkin Elmer Spinning Disk. SOCE inhibition using SKF-963656 (50 μM) dissolved in water was performed on dechorionated embryos using untreated embryos as control. The effect of SKF-96365 treatment on EC proliferation and migration (Supplementary Fig. 14) was performed using continuous exposure to compound within a light sheet chamber using SKF-96365 at a concentration of 50 μM within 0.7% agarose mounting medium and surrounding E3 medium + Tricaine. Embryos were treated from 21 hpf and imaged between 24 and 30 hpf. One to two embryos were imaged for each individual condition and three congruent repeats of each condition were performed.

### Calculation of DA blood flow velocimetry

Single *Z*-plane time lapses of 3 dpf zebrafish DA were acquired at 66 fps using a Zeiss Lightsheet Z.1 microscope. The resulting images were subjected to axial line scan particle image velocimetry designed for Lightsheet imaging using custom authored scripts in MATLAB 2017b®. Each time lapse was analysed frame by frame using intensity-based thresholding of circulating erythrocytes. Thresholded images were made binary followed by conversion of two-dimensional image to one-dimensional (1D) signal by summing up in ‘*y*’ direction (all column pixels). The 1D signal was low pass filtered to remove spurious and highly correlated pixels (low correlation implies displacement of 1D wave signal). Cross-correlation across time was calculated on filtered images for each time frame using inbuilt MATLAB 2017b® functions. Thus, the obtained correlation (in pixels/frames) was then converted to mm/s using the following equation:

$$\rm Velocity(mm/s) = \frac{{Correlation(pixels/frame) \times fps \times 10^{ - 3}}}{{Scale(pixels/\mu m)}}$$

Algorithm is available on request.

### siRNA-TMEM33 knockdown in HUVEC

Small interfering RNA (siRNA)-mediated knockdown of *TMEM33* gene expression in HUVEC cells was performed using ON-TARGET-plus SMART pool human TMEM33 (Thermo Scientific). ON-TARGET-plus non-targeting pool control duplexes (Thermo Scientific) were used as a control. For siRNA transfection, HUVECs (Cellworks, ZHC-2301) were plated in 0.1% gelatin pre-coated six-well tissue culture plates (3 × 10^5^ cells/well) and incubated overnight. The following morning, cells were washed with phosphate-buffered saline and incubated in serum-free, antibiotic-free OPTIMEM medium for 1 h. Then, 100 pmol of siRNA/well were transfected using 5 μl of Lipofectamine 2000 (Thermo Scientific). Transfection medium was removed after incubation for 6 h and substituted by ECGM supplemented with ECGM-supplement mix and 1% penicillin/streptomycin/amphotericin B (Sigma-Aldrich). Cells were incubated for 72 h and then used for further experiments.

### Wound-healing EC migration assay

Cell migration of HUVECs and MLECs, treated as indicated, was assayed using the Cytoselect™ 24-well Wound Healing Assay kit (Cell Biolabs). Images were captured with a Nikon DSFi1 digital camera coupled to a Nikon ECLIPSE TS100 microscope at × 4 magnification. Cell-free area was quantified with ImageJ software.

### Matrigel EC tube-formation assay

HUVECs transfected with the corresponding siRNA were plated onto Geltrex Reduced Growth-Factor Matrix (Invitrogen) in Medium 200 with no phenol red and supplemented with 2% foetal calf serum and VEGF (25 ng/ml) when indicated. Tube formation was quantified after incubation at 37 °C for 16 h. Images were recorded with a Nikon DSFi1 digital camera coupled to a Nikon ECLIPSE TS100 microscope at 4× magnification.

### Statistical analysis

All statistical analysis employed two-tailed tests and are described in figure legends. All error bars display the mean and SD, except for qPCR error bars, which display the mean and SEM. Numbers of experimental repeats are listed in figure legends. *P*-values, unless exact value is listed, are as follows: * < 0.05, ** < 0.01, *** < 0.001 and **** < 0.0001. *F*-values, *t*-values and degrees of freedom are listed in individual figure legends.

### Reporting Summary

Further information on experimental design is available in the Nature Research Reporting Summary linked to this Article.

## Supplementary Information

Supplementary Information

Description of Additional Supplementary Files

Supplementary Movie 1

Supplementary Movie 2

Supplementary Movie 3

Supplementary Movie 4

Supplementary Movie 5

Supplementary Movie 6

Supplementary Movie 7

Supplementary Movie 8

Supplementary Movie 9

Supplementary Movie 10

Supplementary Movie 11

Source Data

Reporting Summary

## Data Availability

The datasets generated during and/or analysed during the current study are available from the corresponding author on reasonable request. The source data underlying Figs. 1l–m, 2b, d, e, h, i, 3g, j, m, p, 4e, h, i, 5a–c, 6c, f–h, u and 7c, f, g, j, m, p, and Supplementary Figs. 2e, 3d, 4d, e, 5b, d, f, h–j, 6c, f, 7e, h, l, o, 8c , e, g, h, k–m, 9j, k, 10k, l , 12c, f , 13c, f, g, j, m and 14e–h are provided as a Source Data file. A Reporting Summary for this Article is available as a Supplementary Information file.
