# Supplementary material for: tmem33 is essential for VEGF-mediated endothelial calcium oscillations and angiogenesis
Source: Nat Commun. 2019 Feb 13;10:732. doi: 10.1038/s41467-019-08590-7 (PMC6374405; doi:10.1038/s41467-019-08590-7)
Supplement: Supplementary file 1 — Supplementary Information [file 41467_2019_8590_MOESM1_ESM.pdf]

**Supplementary Information**

***tmem33* is essential for VEGF-mediated endothelial calcium oscillations and angiogenesis**

**Savage et al.,**

**Supplementary Table 1. Primer sequences**

| **Primer name** | **Sequence (5'-3')** |
|---|---|
| tmem33ex3F | GCCTCAAGCTGCTGCTAACT |
| Tmem33ex3R | TGATGGGGTAGGAGTTGACC |
| tmem33 MO F | CAGACACCGAGCAAAGGAGT |
| tmem33 MO R | TGTAGTGGCGTGGAGAAGTG |
| tmem33 gRNA F * | TTTGTGCAATCCAGAGGTCG |
| tmem33 gRNA R * | TGCGCATAATAACATAGTCAAA |
| LifeAct-CloverF | GAATTCATGGGCGTGGCCGACTTGATCAAGAAGTTCGAGT CCATCTCCAAGGAGGAGGGGGATCCACCGGTCGCCACCAT GGTGAGCAAGGGCGAG |
| Clover-XhoIR | CTCGAGTTACTTGTACAGCTCGTCC |
| LACloverattB1F | GGGGACAAGTTTGTACAAAAAAGCAGGCTTCATGGGCGTG GCCGACTTG |
| LACloverattB2R | GGGGACCACTTTGTACAAGAAAGCTGGTCTTACTTGTACA GCTCGTCC |
| Actinvhh-attb1R | GGGGACAAGTTTGTACAAAAAAGCAGGCTTCATGGCTCAG GTGCAGCTGG |
| Tagrfp-attb2R | GGGGACCACTTTGTACAAGAAAGCTGGGTCTCAATTAAGT TTGTGCCC |

* tmem33 gRNAF/R amplify a 965bp fragment including exon1, intron1, exon2 and were employed to test binding efficacy of each sgRNA using Cas9 protein.

**Supplementary Table 2. gRNA and morpholino sequences (5'-3)**

| | |
|---|---|
| Control morpholino | CCTCTTACCTCAGTTACAATTTATA |
| tmem33 sp2b morpholino | AATTGCTAACACAGACATGACAA |
| tmem33 sp3 morpholino | TTATAGGAGAACATGACTCACCCAA |
| ***Dll4*** gRNA | AAAGCACCGACTCGGTGCCACTTTTTCAAGTTGATAACGGACTAGCCTTATTTTAACTTGCTATTTCTAGCTCTAAAAC**AATCGAAAAGCATGGCAGCT**CTATAGTGAGTCGTATTACGC |
| ***tmem33*** 5'utr gRNA | AAAGCACCGACTCGGTGCCACTTTTTCAAGTTGATAACGGACTAGCCTTATTTTAACTTGCTATTTCTAGCTCTAAAAC**GAGGTCGGCTGGCTCAGCTGACC**CTATAGTGAGTCGTATTACGC |
| ***tmem33*** ex1 gRNA | AAAGCACCGACTCGGTGCCACTTTTTCAAGTTGATAACGGACTAGCCTTATTTTAACTTGCTATTTCTAGCTCTAAAAC**TCCTCCTCCTCCTCAGGCTGGGGC**TATAGTGAGTCGTATTACGC |
| ***tmem33*** 5'utr ctrl gRNA | AAAGCACCGACTCGGTGCCAC**GAGGTCGGCTGGCTCAGCTGACC**CTATAGTGAGTCGTATTACGC |
| ***tmem33*** ex1 ctrl gRNA | AAAGCACCGACTCGGTGCCAC**TCCTCCTCCTCCTCAGGCTGGGGC**TATAGTGAGTCGTATTACGC |

**Supplementary Table 3. Taqman probes**

| | |
|---|---|
| *eef1a* | Dr03432748_m1 |
| *flt1* | Dr03109249_m1 |
| *kdr* | Dr03116242_m1 |
| *flt4* | Dr03138049_m1 |
| *kdrl* | Dr03432904_m1 |
| *tmem33* | Dr03140164_m1 |
| *p53* | Dr03112089_m1 |
| *notch1b* | Dr03086775_m1 |
| *dll4* | Dr03428646_m1 |
| *her12* | Dr03133031_m1 |
| *hey2* | Target sequence: GGAATGAAGTTTGAGACCTCCATTCGACGGCTCGGGGCGTGTTTTCTATTTTTTTTTTACGGTGGGTGTTCCCGAAGCAGGACGTGGGCGTGAATGTGAGACTGAGGCTCCAGCGGTTCGTGGGAAAGGCGCTCAGAGAGTTTTTGGTGTCTGTACCTGCGCGCACTGCATCATGAAGCGGCCCTGTGAGGACAGCACGTCCGACAGCGACATGGATGAAACCATTGATGTGGGCAGCGAGAATAACTACTCTGGCCAAAGCAATGGTTCATTTATAAGATGTGGCTCACCTACAACGACATCCCAAGTCATGGCCAGAAAGAAGCGGAGAGG |

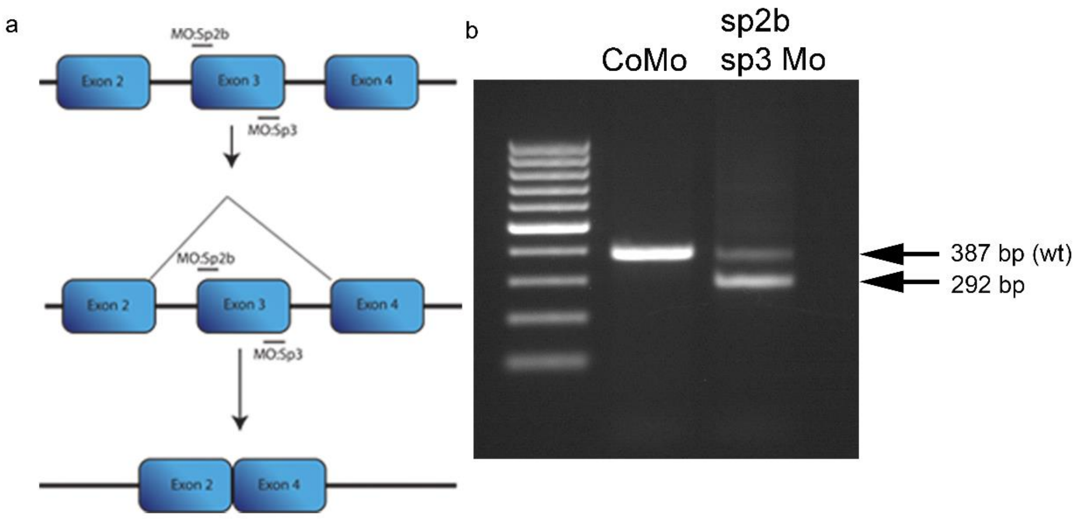


**Supplementary Fig. 1** ***tmem33*** **morpholinos individually impair SeA formation. a**) Schematic representation of *tmem33* splice blocking morpholinos. Sp2b and Sp3 target *tmem33* mRNA flanking exon 3. **b**) Co-injection of *tmem33* morpholinos (0.4ng total) induce a 95 bp deletion as indicated by RT-PCR. This corresponds to exon3 and induces mis-splicing resulting in skipping of exon 3 which leads to a frameshift and premature stop codon within exon 4.

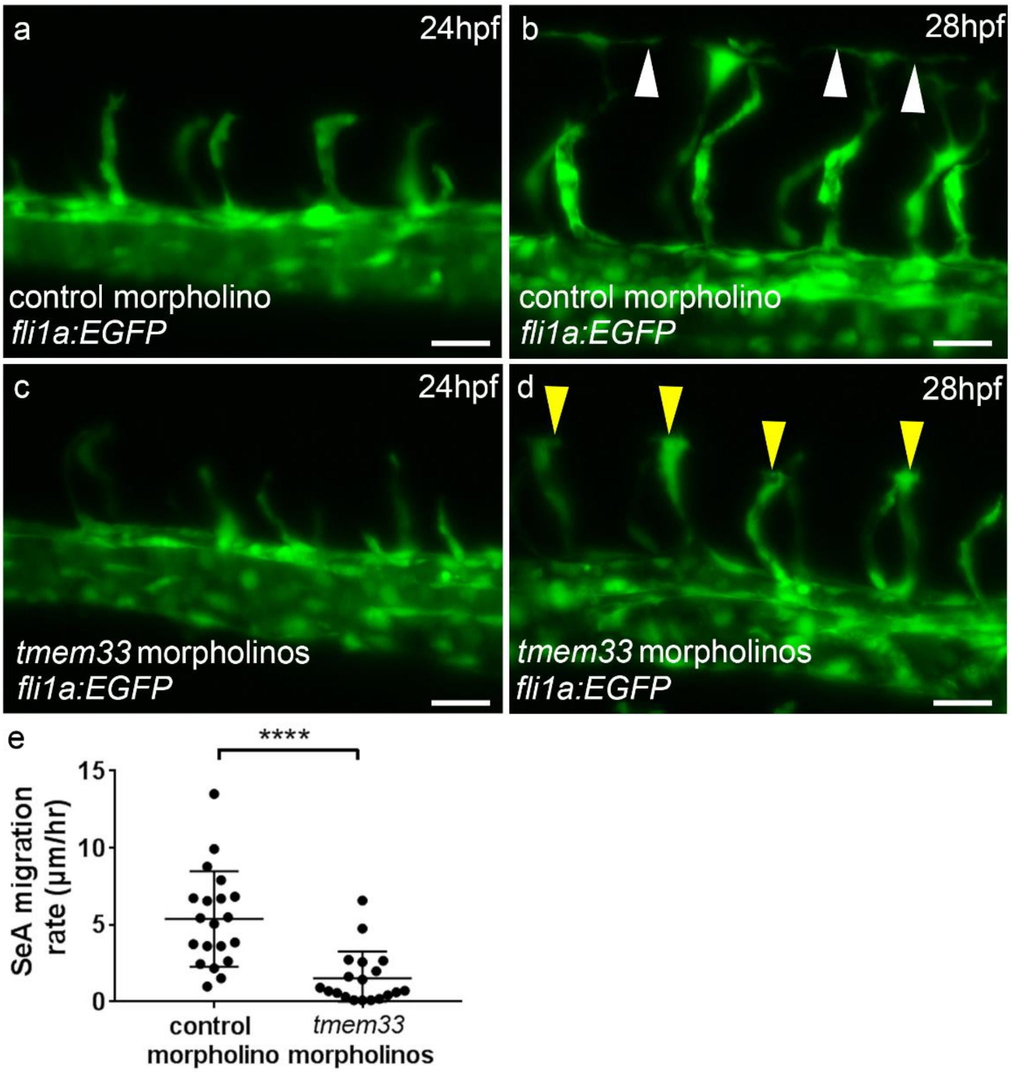


**Supplementary Figure 2. *tmem33* morphants display reduced segmental artery migration. a-d**) Timelapse confocal images were taken between 24hpf and 28hpf. Control morphants (0.4ng) (a, b) display normal segmental artery migration and begin to anastomose to form the DLAV 4 hours after start of imaging (**b**, white arrowheads). *tmem33* morphants (0.4ng) (c, d) display reduced segmental artery migration compared to control morphants and SeAs fail to complete dorsal migration or begin DLAV anastomosis (**d**, yellow arrowheads) by 4.5 hours after start of imaging. **e**) *tmem33* morphants display reduced rate of segmental artery migration between 24-28hpf (Mann-Whitney U-test, ****p=<0.0001; 2 repeats; n=10 embryos per group). Scale bars 50 µm. Source data are provided as a Source Data file.

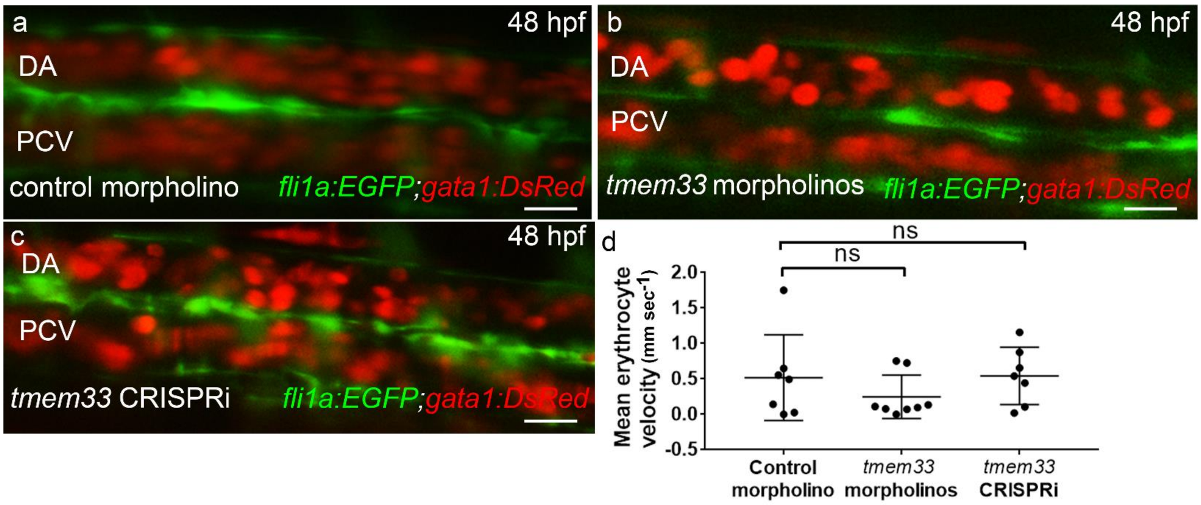


**Supplementary Figure 3. Aortic erythrocyte velocity is not impaired following *tmem33* knockdown. a-c**) Control morphants (0.4ng) (**a,** Supplementary movie 1)**,** *tmem33* morphants (0.4ng) (**b,** Supplementary Movie 2) and *tmem33* global crispants (**c,** Supplementary Movie 3) display circulating erythrocytes (red cells) within the dorsal aorta (DA) and posterior cardinal vein (PCV). Still images from corresponding supplementary movie are shown. **d**) No significant difference in mean erythrocyte velocity is observed between control morphants, *tmem33* morphants or *tmem33* crispants (One-way ANOVA ns=not significant; F=1.03; FD=0.263, DF =3; 2 repeats; n=3 or 4 embryos per group). Scale bars 10µm. Source data are provided as a Source Data file.

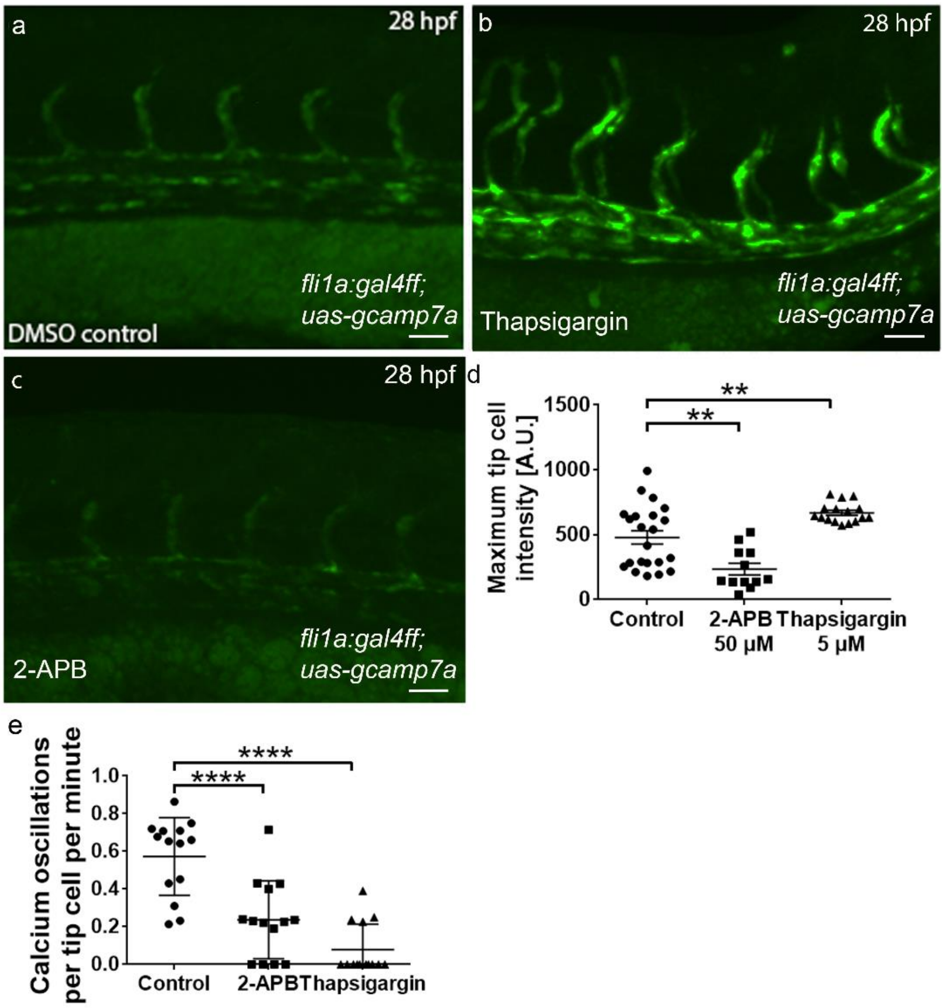


**Supplementary Figure 4. *fli1a:gal4ff;uas-gcamp7a* embryos report modulation of endothelial $Ca^{2+}$ signalling *in vivo.*** **a**) *Tg(fli1a:gal4ff;uas-gcamp7a)* embryos display EC GCaMP7a fluorescence. **b**) Thapsigargin treatment (5µM) for 45 minutes increases EC GCaMP7a fluorescence in *Tg(fli1a:gal4ff;uas-gcamp7a)* embryos. **c**) 2-APB reduces GCaMP7a fluorescence in *Tg(fli1a:gal4ff;uas-gcamp7a)* embryos. **d**) 2-APB and thapsigargin modulate GCaMP7a fluorescence in endothelial cells, with 2-APB reducing GCaMP7a fluorescence and thapsigargin increasing fluorescence (One-way ANOVA **p=<0.01; F=19.43 DF=2, 3 repeats, n=4-6 embryos per group). **e**) 2-APB and thapsigargin reduce $Ca^{2+}$ oscillation frequency in endothelial tip cells (One-way ANOVA *p=<0.05, **p=<0.01; F=25.88; DF=2; 3 repeats; n=≥4 embryos per group). Scale bars 50 µm. Source data are provided as a Source Data file.

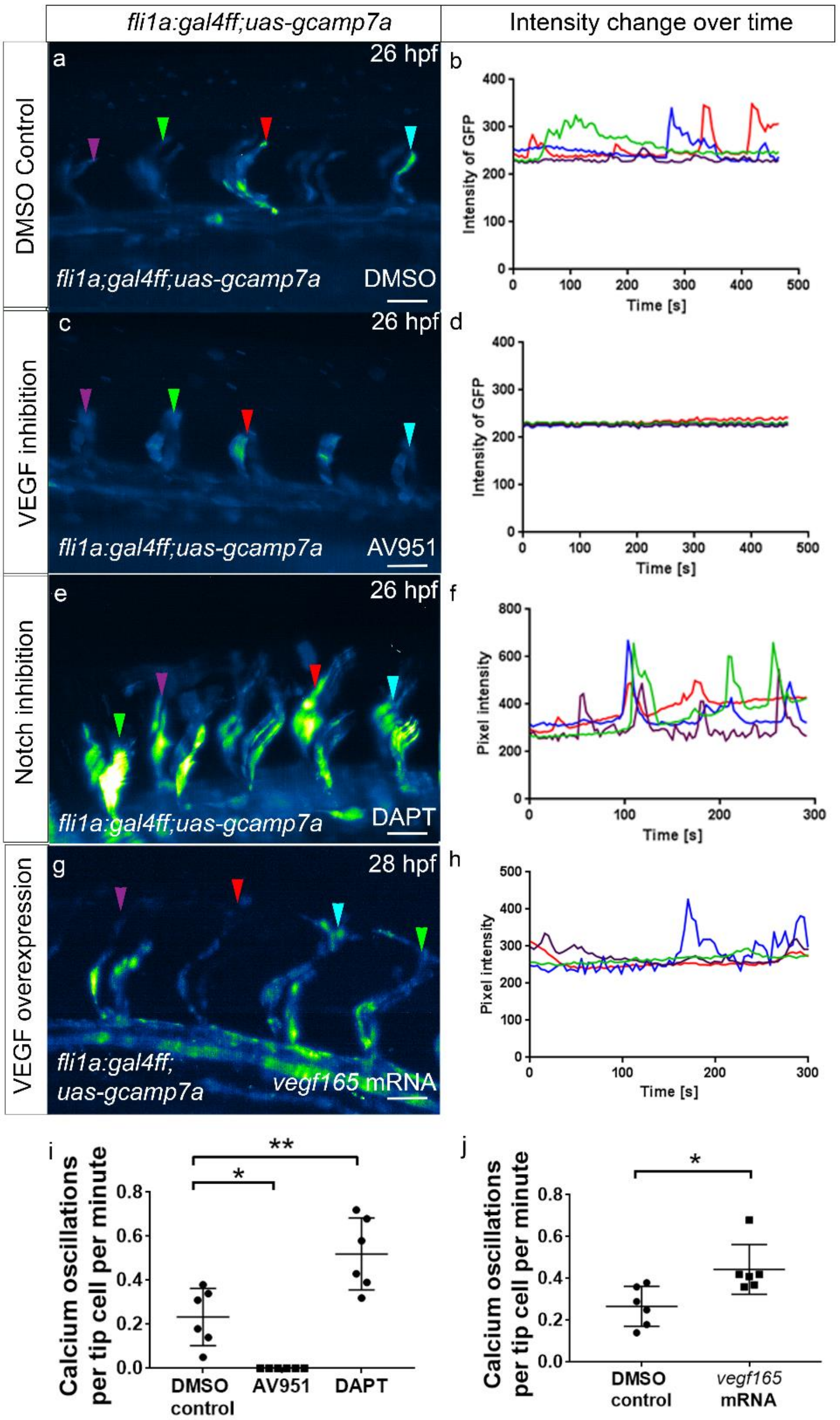


**Supplementary Figure 5. Modulation of VEGF and Notch signalling modify frequency of endothelial $Ca^{2+}$ oscillations. a**) Fluorescence intensity projections over time are shown. Control *Tg(fli1a:gal4ff;uas-gcamp7a)* embryos display changes in GCaMP7a fluorescence intensity over time in ECs. **b**) Change in fluorescence plotted against time in n=4 EC tip cells

in control embryos. Coloured traces correspond to arrowheads in (**a**). **c**) *Tg(fli1a:gal4ff;uas-gcamp7a)* embryos treated with 500nM AV951 treatment abrogates $Ca^{2+}$ oscillations in EC tip cells in representative *Tg(fli1a:gal4ff;uas-gcamp7a)* embryo. **d**) Change in GCaMP7a fluorescence intensity over time in in n=4 EC tip cells in representative AV951-treated embryo. Coloured traces correspond to arrowheads in (**c**). **e**) DAPT treatment increases GCaMP7a fluorescence intensity over time and induces ectopic $Ca^{2+}$ oscillations in stalk cells. Image corrected for sample movement. **f**) Change in GCaMP7a fluorescence over time in n=4 EC tip cells in representative DAPT-treated embryo. Coloured traces correspond to arrowheads in (**e**). **g**) Overexpression of *vegfa*$_{165}$ mRNA (400pg) increases GCaMP7a fluorescence intensity and ectopic $Ca^{2+}$ oscillations in stalk cells and the DA in *Tg(fli1a:gal4ff;uas-gcamp7a)* embryos. **h**) Change in fluorescence over time in n=4 EC tip cells in representative following *vegfa*$_{165}$ overexpression. Coloured traces correspond to arrowheads in (**g**). **i**) Frequency of $Ca^{2+}$ oscillations per tip cell per minute is significantly reduced by AV951 treatment and significantly increased by DAPT treatment (One-Way ANOVA, *p=<0.05, **p=<0.01; F= 27.99, DF=2, 2 repeats; n= 3 embryos per group). **j**) Overexpression of *vegfa*$_{165}$ significantly increases $Ca^{2+}$ oscillation frequency per tip cell per minute (unpaired t-test, *p=<0.05; t=2.835 DF=10; 2 repeats; n= 3 embryos per group). Scale bars 50 µm. Source data are provided as a Source Data file.

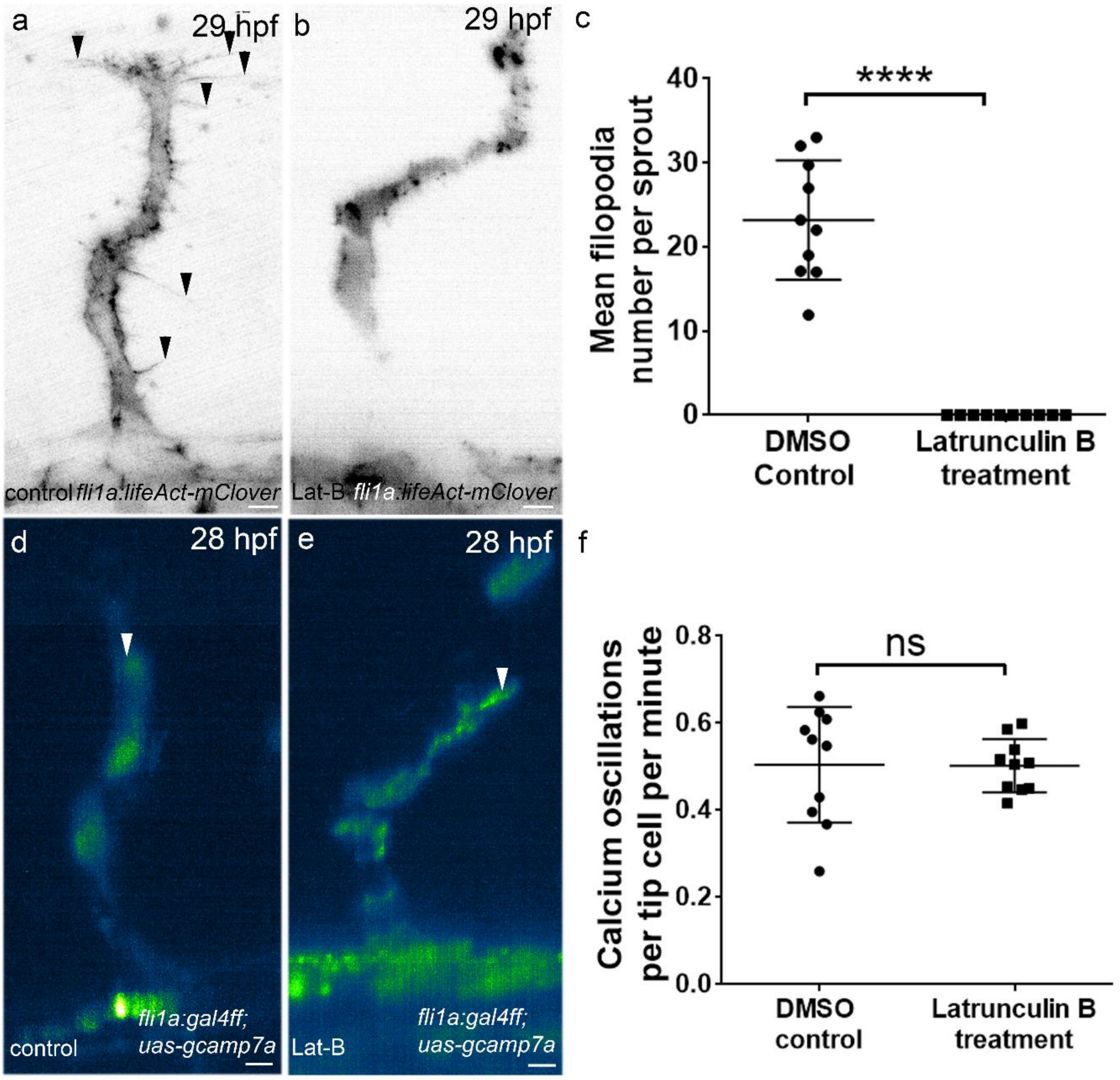


**Supplementary Figure 6. Endothelial $Ca^{2+}$oscillations within tip cells are not disrupted following loss of filopodia. a, b**) Latrunculin B treatment reduces endothelial filopodia number in *Tg(fli1a:lifeact-mClover)* embryos (black arrowheads). **c**) Latrunculin B treated *Tg(fli1a:lifeact-mClover)* embryos display significant reductions of filopodia number per SeA (unpaired t-test ****p=<0.0001; t=10.34; DF=14; 2 repeats; n= 5 embryos per group). **d-f**) Frequency of $Ca^{2+}$ oscillations in tip cells (arrowheads) are not significantly altered by Latrunculin B treatment in *Tg(fli1a:gal4ff;uas-gcamp7a)* embryos (Mann-Whitney U-test, non significant; 2 repeats; n= 5 embryos per group). Scale bars 5 µm. Source data are provided as a Source Data file.

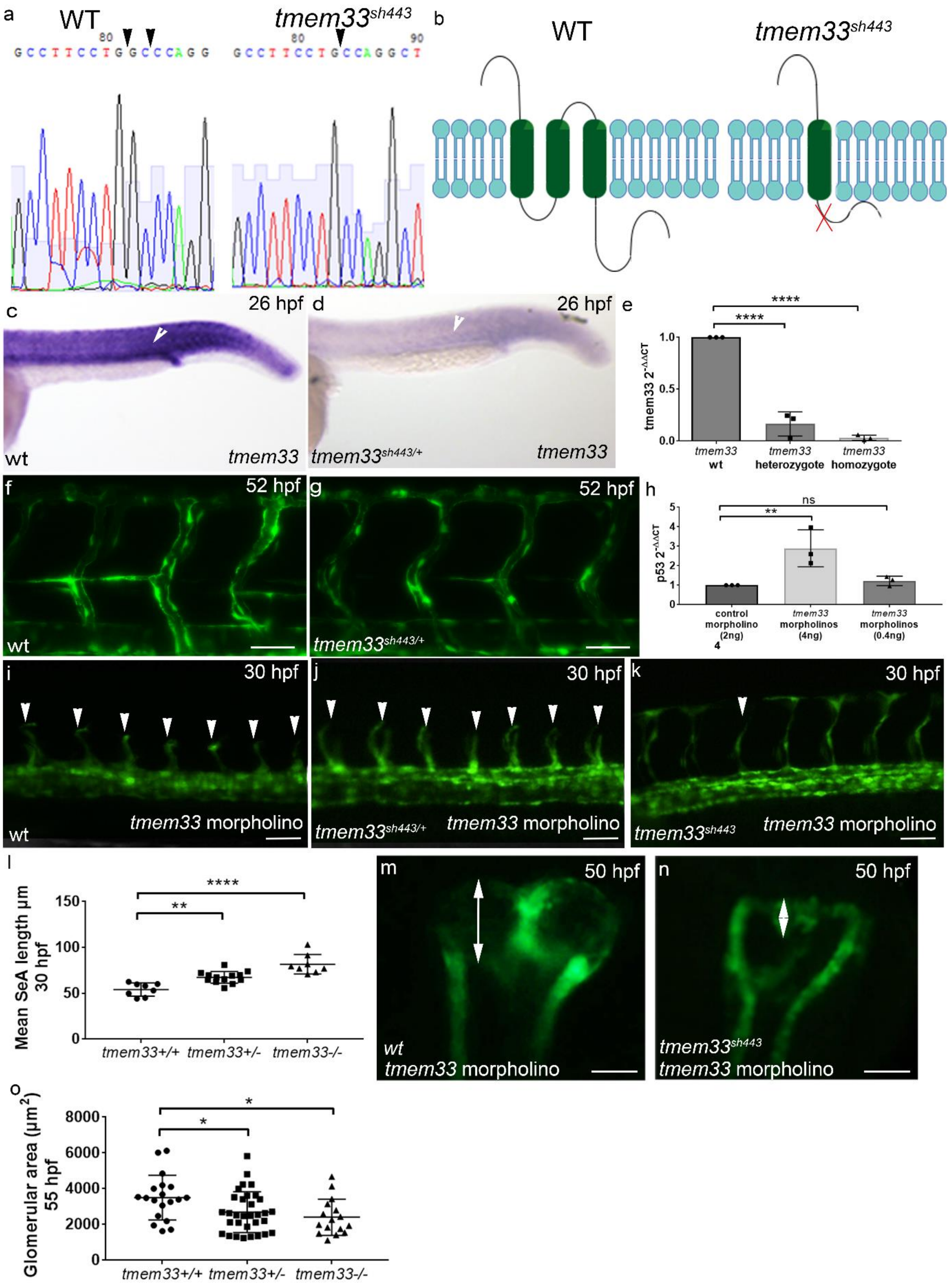


**Supplementary Figure 7. *tmem33*$^{sh443}$ mutant embryos are morphologically normal, display reduced expression of *tmem33* mRNA and are less sensitive to *tmem33* morpholino injections**

**a**) *tmem33*$^{sh443}$ allele consists of a 2bp deletion within exon 3, causing a frameshift. **b**) Schematic representation of predicted mutant protein, highlighting loss of two conserved transmembrane domains. **c, d**) *tmem33* expression is reduced in *tmem33*$^{sh443}$ mutants (D) compared to wild type siblings, (c, white arrowhead). **e**) *tmem33* expression is significantly reduced in *tmem33*$^{sh443/+}$ and *tmem33*$^{sh443}$ embryos by qRT-PCR (One-Way ANOVA, ****p= <0.0001; F=176.1 DF=2. 3 repeats; n=30 embryos per group). **f, g**) *tmem33*$^{sh443}$ mutant *Tg(fli1a:egfp)* embryos display normal vascular morphology at 52 hpf, compared to wild type. Scale bars 50 µm. **h**) Titration of morpholino to dose which does not significantly induce p53 (0.4ng) (One-way ANOVA **p=<0.01, ns = non-significant; F=1.359; DF=38; 3 repeats per group; n= 30 embryos per group). **i**) Representative *tmem33*$^{+/+}$*;Tg(fli1a:egfp)*$^{y1}$ embryo displaying reduced SeA length (white arrowheads) at 30 hpf. **j**) Representative *tmem33*$^{sh443/+}$*;Tg(fli1a:egfp)*$^{y1}$ embryo displaying reduced SeA length (white arrowheads) at 30 hpf. Scale bars 50 µm. **k**) Representative *tmem33*$^{sh443}$*;Tg(fli1a:egfp)*$^{y1}$ embryo displaying normal SeA length (white arrowheads) at 30 hpf. Scale bars 50 µm. **l**) *tmem33*$^{sh443/+}$ *Tg(fli1a:egfp)*$^{y1}$ heterozygous incross injected with 0.4ng *tmem33* morpholinos, blindly ranked for phenotype and retrospectively genotyped. *tmem33*$^{sh443}$ embryos display protection from morphant phenotype (One-way ANOVA with Tukey's multiple corrections: **p=<0.01; ***p=<0.001; F=24.02; DF=0.5135; 2 repeats; *tmem33*$^{+/+}$ n=8, *tmem33*$^{+/-}$ n=13, *tmem33*$^{-/-}$ n=8) **m**) Representative *tmem33*$^{+/+}$*;Tg(wt1b:egfp)*$^{li1}$ embryo displaying glomerular expansion (white arrow) at 50 hpf. Scale bars 200 µm. **n**) Representative *tmem33*$^{sh443}$*;Tg(wt1b:egfp)*$^{li}$ embryo displaying normal glomeruli (white arrow) at 50 hpf. Scale bars 200 µm. **o**) *tmem33*$^{sh443/+}$ *Tg(wt1b:egfp)*$^{li1}$ heterozygous incross injected with 0.4ng *tmem33* morpholinos, blindly ranked for phenotype and retrospectively genotyped (One-way ANOVA with Tukey's multiple corrections *p=<0.05; F=8.26 DF=2.751; 2 repeats; *tmem33*$^{+/+}$ n=19 *tmem33*$^{+/-}$ n=32, *tmem33*$^{-/-}$ n=16). Source data are provided as a Source Data file.

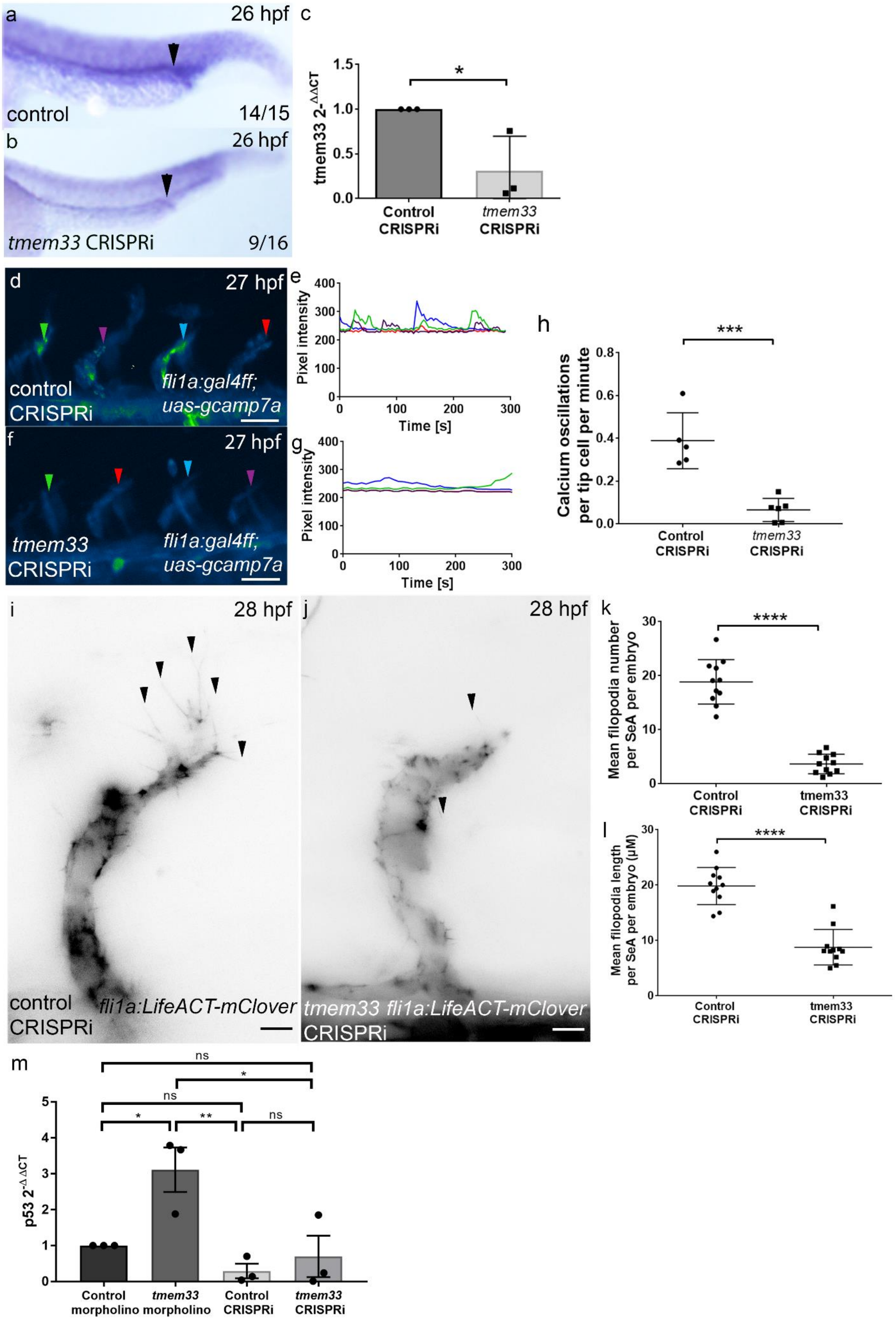


**Supplementary Figure 8.** ***tmem33*** **CRISPRi recapitulates** ***tmem33*** **morpholino phenotypes**

**a, b**) *tmem33*CRISPRi injected embryos display reduced *tmem33* expression by *in situ* hybridisation (black arrowhead indicates developing pronephros). **c**) *tmem33* expression is significantly reduced by CRISPRi (n=3 repeats, unpaired t-test *p=<0.05; t=5.861 DF=8). **d-g**) *tmem33* CRISPRi reduces frequency of tip cell $Ca^{2+}$ oscillations in *Tg(fli1a:gal4ff;uas-gcamp7a)* embryos. Intensity projection over time (**d, f**) depicting regions GCaMP7a fluorescence intensity over time-lapse (arrowheads). Scale bars 50 µm. **h**) *tmem33* CRISPRi significantly reduces the frequency of $Ca^{2+}$ oscillations per tip cell per minute (unpaired t-test, ***p=<0.001; t=5.559 DF=9, n=5 or 6 embryos per group). **i, j**) *tmem33* crispants display reduced filopodia number per SeA in *Tg(fli1a:LifeACT-mClover*) embryos (black arrowheads) compared to controls. Scale bars 5 µm.  **k**) *tmem33* CRISPRi significantly reduces filopodia number (unpaired t-test ****p=<0.0001; t=11.22; DF=20; 2 repeats; n=5 or 6 embryos per group). **l**) *tmem33* CRISPRi significantly reduces filopodia length (unpaired t-test ****p=<0.0001; t=7.902; DF=20; 2 repeats; n=5 or 6 embryos per group). **m)** Induction of *p53* expression is reduced in *tmem33* crispants in comparison to *tmem33* morphants (4ng). All values were normalised relative to *eef1a* (n= 3 repeats, 20 embryos per condition, one-way ANOVA, Tukey’s multiple comparison, *p=<0.05; **p=<0.01; F=8.35, DF=3). Source data are provided as a Source Data file.

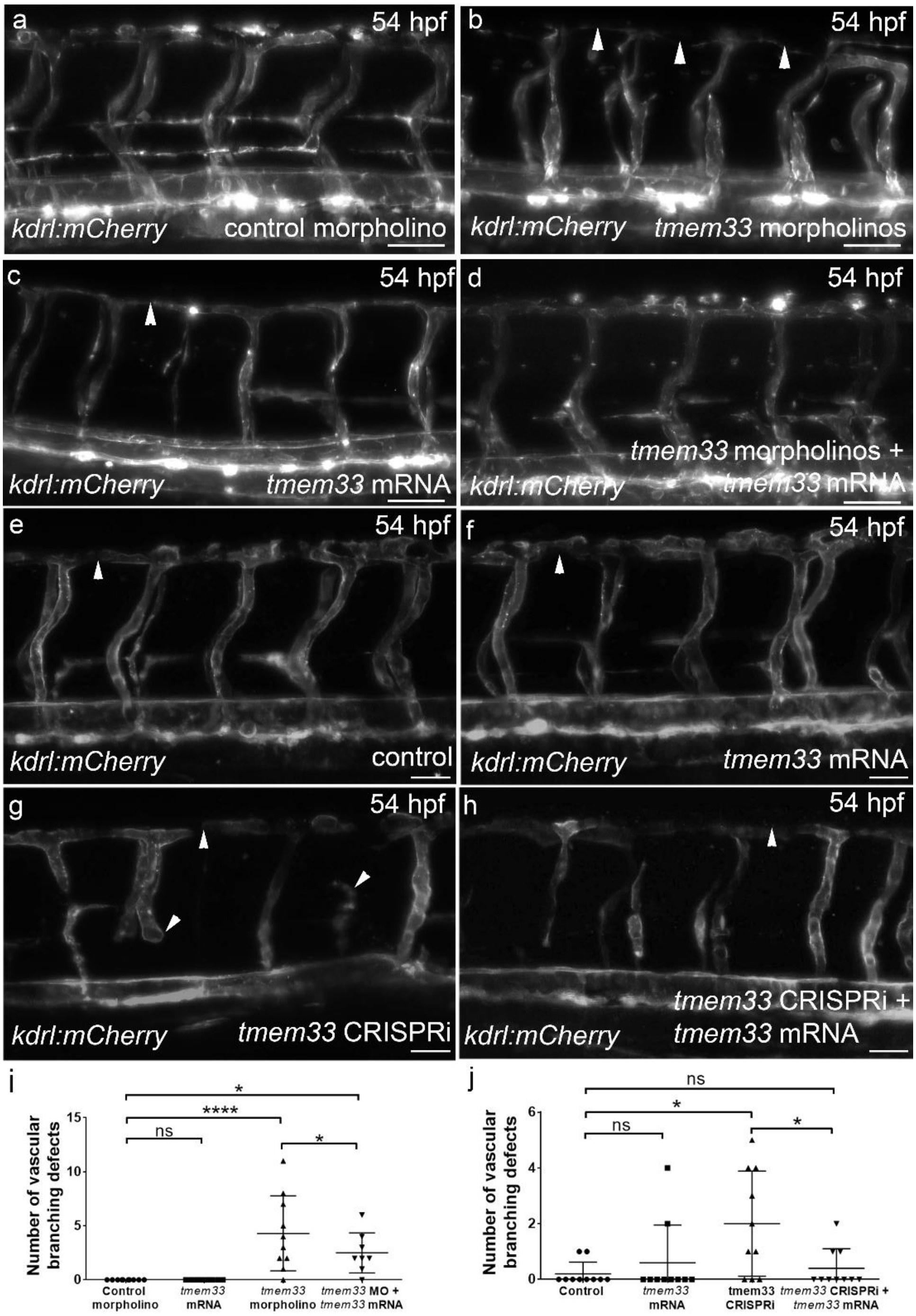


**Supplementary Figure 9. *tmem33* mRNA partially rescues angiogenic defects in *tmem33* crispants.**

**a**) Control morphant *Tg(kdrl:mCherry)* embryos (0.4ng) display normal vascular development at 54 hpf, including normal DLAV development (white arrowhead). **b**) Overexpression of full length *tmem33* mRNA does not impair vascular development (white arrowhead). **c**) *tmem33* morphants (0.4ng) display vascular developmental defects, including misbranching and

incomplete DLAV formation (white arrowheads). **d**) Representative embryo following simultaneous *tmem33* mRNA overexpression and *tmem33* morpholino knockdown (0.4ng) (white arrowhead). **e**) Control morpholino injected *Tg(kdrl:mCherry)* embryos (0.4ng) display normal vascular development at 54 hpf, including normal DLAV development (white arrowhead). **f**) Overexpression of full length *tmem33* mRNA does not impair vascular development (white arrowhead). **g**) *tmem33* CRISPRi induces vascular developmental defects, including misbranching and incomplete DLAV formation (white arrowheads). **h**) Representative embryo following simultaneous *tmem33* mRNA overexpression and *tmem33* CRISPRi (white arrowhead). **i**) Overexpression of full length *tmem33* mRNA reduces frequency of angiogenic defects in *tmem33* morphants (One way ANOVA with Dunnett's corrections *p=<0.05; F=11.19, DF=10.16. 2 repeats; n=5 embryos per group). **j**) Overexpression of full length *tmem33* mRNA reduces frequency of angiogenic defects following *tmem33* CRISPRi (One way ANOVA with Dunnett's corrections *p=<0.05; F=4.412, DF=3. 2 repeats; n=5 embryos per group). All scale bars 50 µm. Source data are provided as a Source Data file.

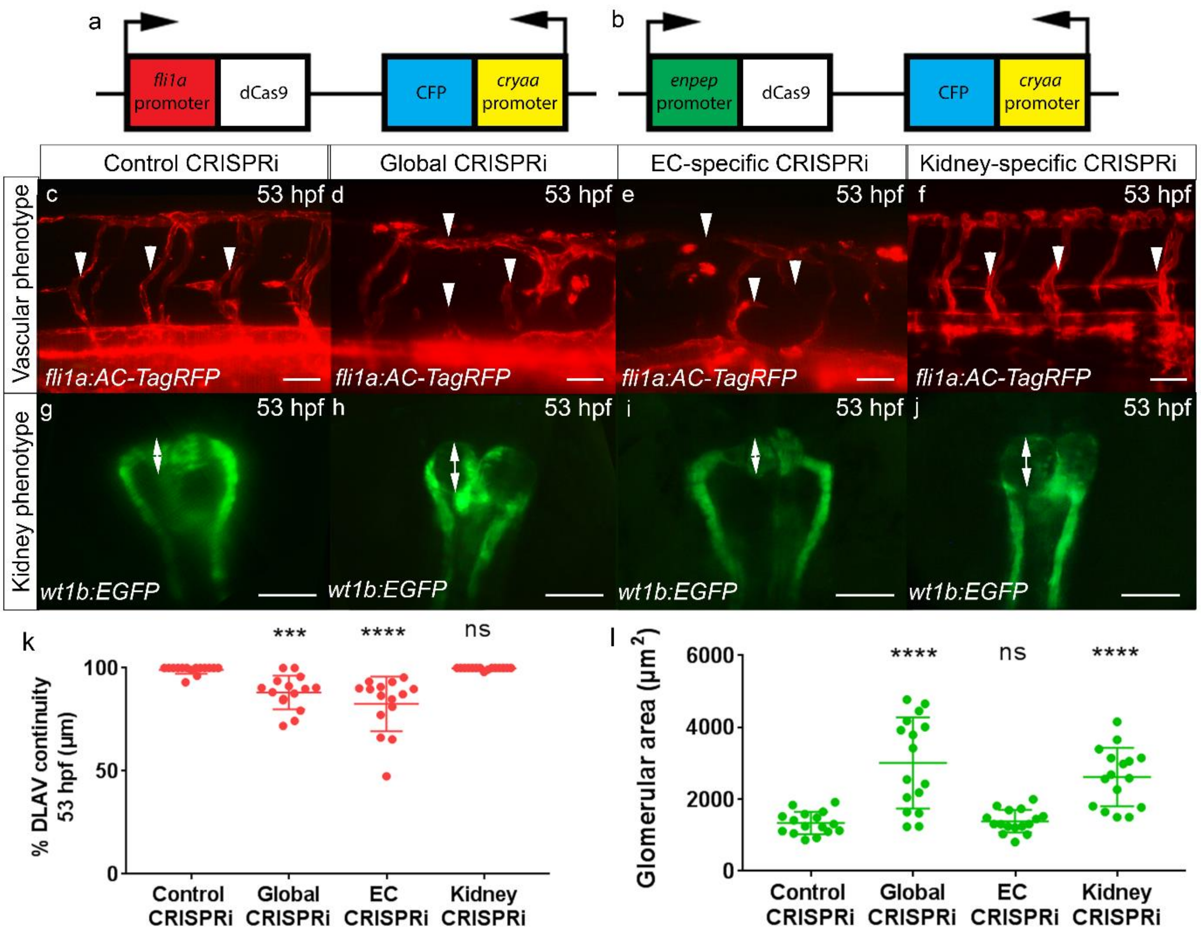


**Supplementary Figure 10.** ***tmem33*** **promotes angiogenesis in an EC-specific manner. a**) Schematic representation of *fli1a:dCas9*, *cryaa:CFP* construct for transient EC specific expression of dCas9 and (**b**) *enpep:dCas9, cryaa:CFP* construct for pronephric-specific expression of dCas9. **c-f**) Both global and endothelial-specific *tmem33* knockdown by CRISPRi inhibit DLAV formation in *Tg(fli1a:AC-TagRFP;wt1b:egfp)* embryos, while global control CRISPRi and pronephric-specific CRISPRi do not (Scale bars 50 µm). **g-j**) Both global and *tmem33* pronephric-specific knockdown CRISPRi knockdown increase glomerular size in *Tg(fli1a:AC-TagRFP;wt1b:egfp)* embryos, while global control CRISPRi and endothelial-specific CRISPRi do not (Scale bars 200 µm). **k**) DLAV formation is impaired by global knockdown of *tmem33* by CRISPRi, EC-specific *tmem33* knockdown by CRISPRi, but not by pronephric-specific CRISPRi (One-way ANOVA with Dunnett’s corrections ****p=<0.0001; F=17.25, DF=6.98 2 repeats, n= 7 or 8 embryos per group). **l**) Glomerular size is significantly

increased following global knockdown of *tmem33* by CRISPRi and pronephric-specific *tmem33* knockdown by CRISPRi, but not by EC-specific CRISPRi (Kruskal-Wallace test with Dunn's corrections ****p=<0.0001; F=81.78, DF=2. 2 repeats, n=8 embryos per group). Source data are provided as a Source Data file.

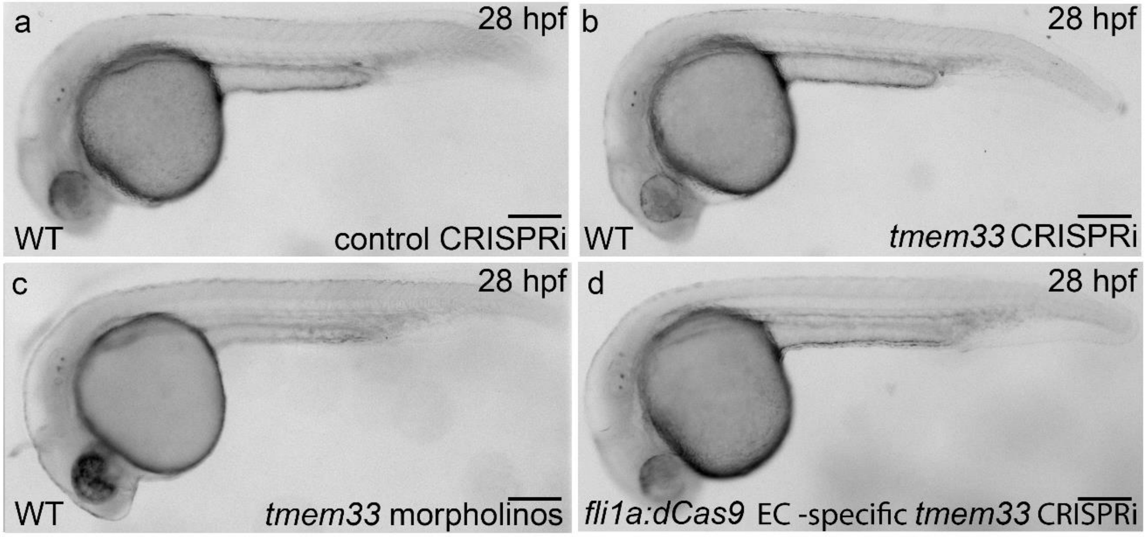


**Supplementary Figure 11. *tmem33* knockdown does not induce gross morphological defects.** Representative image of a control crispant (**a**), *tmem33* crispant (**b**), *tmem33* morphant (0.4ng) (**c**) and EC-specific *tmem33* crispant (**d**) at 28 hpf, illustrating normal gross morphology. Scale bars 250 µm.

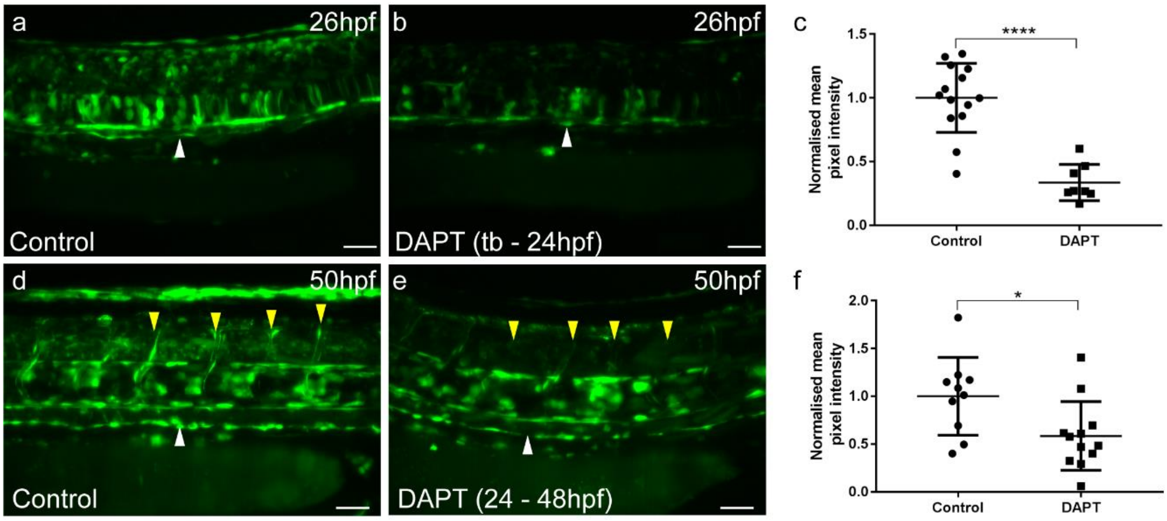


**Supplementary Figure 12. *Tg(CSLBS:Venus)*$^{qmc61Tg}$ reliably reports Notch signalling activity within the developing vasculature. a-c)** Treatment of *Tg(CSLBS:Venus)*$^{qmc61Tg}$ embryos with 100µM DAPT between tailbud and 24hpf, reduces Venus fluorescence within the DA (b, white arrowheads) in comparison to controls (a) (Unpaired t-test **** p=<0.0001, t=6.397, DF =20 n=14 control embryos, n=8 treatment embryos). **d-f)** Treatment of *Tg(CSLBS:Venus)*$^{qmc61Tg}$ embryos with 100µM DAPT from 24 hpf until 48hpf (e), reduces Venus fluorescence within the DA (white arrowheads) (Unpaired t-test * p=0.0194, t= 2.542 DF= 20 n=10 control embryos, n=12 treatment embryos) in comparison to controls (d). Reduced Venus fluorescence in SeAs of DAPT treated embryos is highlighted with yellow arrowheads. To quantify fluorescence, a region of interest was manually selected along the length of the DA, mean pixel intensity was measured, background fluorescence from yolk cell extension was subtracted and each dataset normalised to the mean of each control group. Scale bars 50µm. DA; dorsal aorta. Source data are provided as a Source Data file.

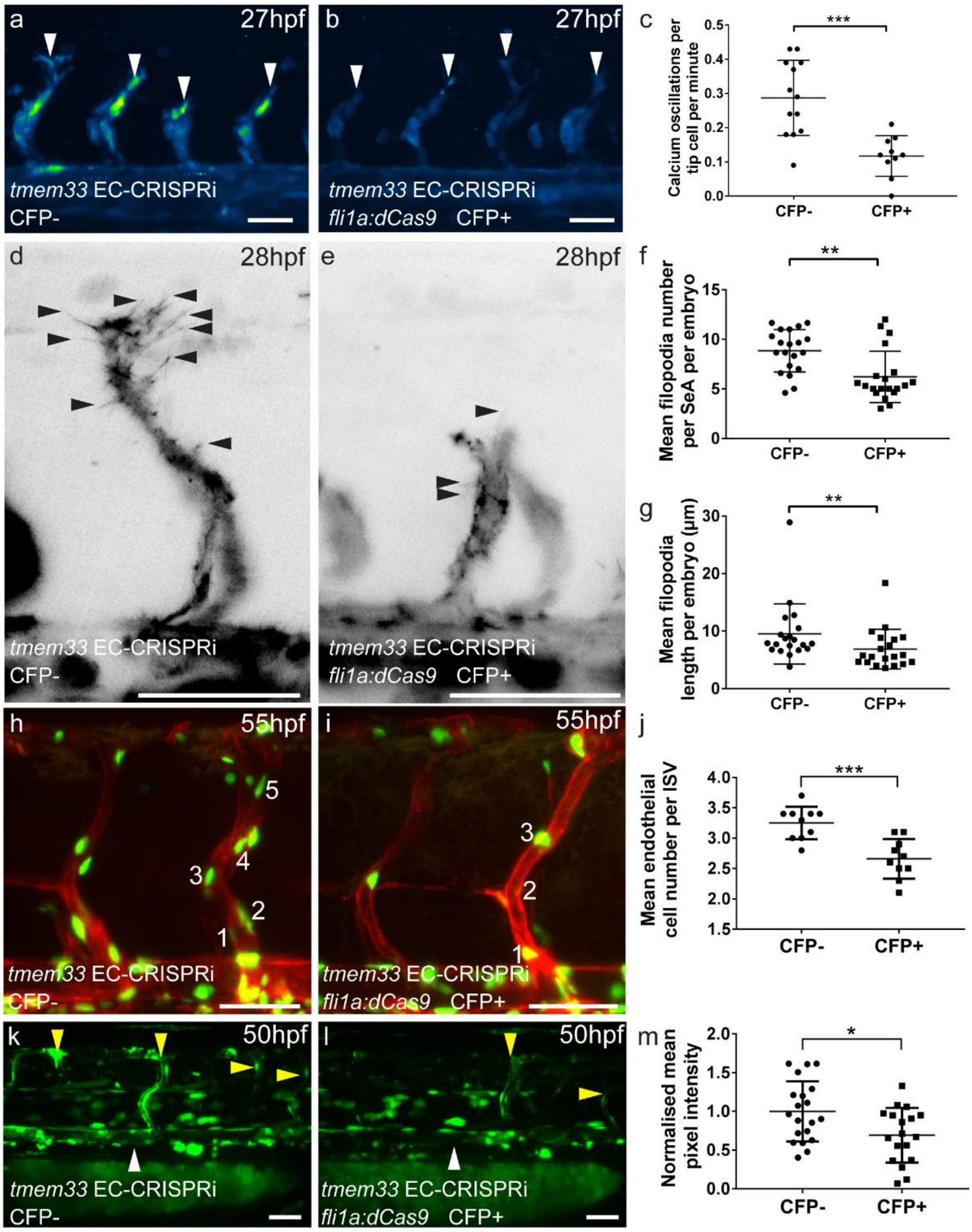


**Supplementary Figure 13. *tmem33* functions cell autonomously to promote angiogenesis. a-c)** EC specific CRISPRi targeting *tmem33* significantly reduced endothelial Ca$^{2+}$ oscillations in a *Tg(fli1a:gal4FF$^{ubs3}$;uas-gcamp7a)* background. Intensity projections show Ca$^{2+}$ oscillations in both internal negative control (CFP-) and following EC specific CRISPRi (CFP+), highlighting intensity over duration of the time-lapse. Change in fluorescence over 300s for four SeAs (**c,** unpaired t-test ***p=0.0003; t=4.399, DF= 21, 2 repeats, n=11 or 12

embryos per repeat). **d-g**) EC specific CRISPRi targeting *tmem33* delayed migration of SeAs which extended fewer filopodia (black arrowheads) in a *Tg(fli1a:lifeACT-mClover)* background compared to controls. Measurements taken from n=4 SeAs per embryo (**f**, unpaired t test **p=0.0011; t=3.519, DF=38, 2 repeats, n=20 embryos per repeat). **g**) EC specific CRISPRi targeting *tmem33* reduced filopodia length compared to controls, measurements taken from n=4 SeAs per embryo (Mann-Whitney U test *p=0.0155, U=111, 2 repeats, n=20 embryos per repeat). **h-j**) EC specific CRISPRi targeting *tmem33* significantly reduced EC number per ISV sprout in a *Tg(kdrl:HRAS-mCherry-CAAX); Tg(flk1:EGFP-NLS)* background. Measurements taken from n=10 ISVs per embryo (**j**, unpaired t-test ***p=0.0003; t=4.413, DF=18, 2 repeats, n=5 embryos per group). **k-m**) EC specific CRISPRi targeting *tmem33* significantly reduced Notch reporter expression within the developing vasculature in a *Tg(csl:venus)* background (SeAs, yellow arrowheads, DA, white arrowheads, **m**, unpaired t-test *p=0.0154; t=2.543, DF=36, 2 repeats, n=9 or 10 embryos per group). Scale bars 50µm. Source data are provided as a Source Data file.

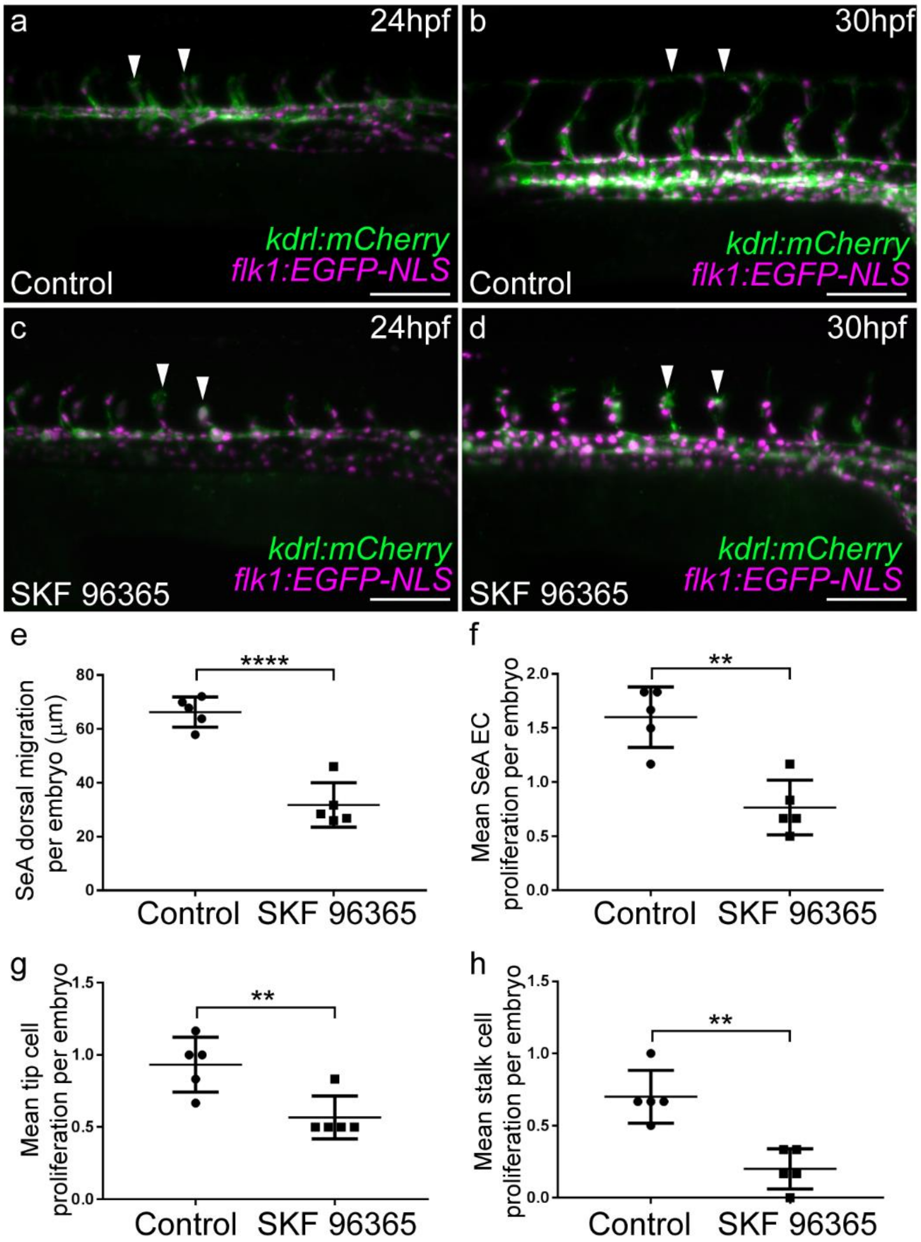


**Supplementary Figure 14. Inhibition of store operated calcium entry impairs EC migration and proliferation during sprouting angiogenesis. a-d**) Embryos were imaged in the presence or absence of SKF-96365 treatment between 24 and 30hpf. Representative images are shown from the start and end point of each time lapse. While control embryos display normal SeA migration and DLAV formation by 30hpf (a, b, arrowheads), SKF-96365 treated embryos display reduced SeA migration, abnormal tip cell morphology and fail to form the DLAV by 30hpf (c, d, arrowheads). **e**) SKF96365-treated embryos display reduced SeA migration, compared to controls (unpaired t-test; ****p=0.0001; t=7.737, DF=8, 3 repeats per

condition). **f**) SKF-96365 treated embryos display reduced EC proliferation per SeA per embryo compared to controls (unpaired t-test; **p=0.0011; t=4.951; DF=8; 3 repeats per condition, 1 or 2 embryos imaged per repeat). **g**) SKF-96365 treated embryos display reduced tip cell proliferation compared to controls (unpaired t-test; **p=0.0094; t=3.395, DF=8, 3 repeats per condition, 1 or 2 embryos imaged per repeat). **h**) SKF-96365 treated embryos display reduced stalk cell proliferation compared to controls (unpaired t-test; **p=0.0012; t=4.867, DF=8, 3 repeats per condition, 1 or 2 embryos imaged per repeat). Source data are provided as a Source Data file.
