# Supplementary material for: tmem33 is essential for VEGF-mediated endothelial calcium oscillations and angiogenesis
Source: Nat Commun. 2019 Feb 13;10:732. doi: 10.1038/s41467-019-08590-7 (PMC6374405; doi:10.1038/s41467-019-08590-7)
Supplement: Supplementary file 2 — Description of Additional Supplementary Files [file 41467_2019_8590_MOESM2_ESM.docx]

**Description of Additional Supplementary Files**

**File Name:** Supplementary Movie 1

**Description:** Circulating erythrocytes in control morphant at 48hpf. Time lapse- recording of 3D rendered lightsheet images of Tg(fli1a:EGFP;gata1a:Dsred) embryo injected with control morpholino displaying circulating erythrocytes within the DA. Elapsed time from the start point of imaging is in seconds, anterior is left, lateral view.

**File Name:** Supplementary Movie 2

**Description:** Circulating erythrocytes in tmem33 morphant at 48hpf Time lapse- recording of 3D rendered lightsheet images of Tg(fli1a:EGFP;gata1a:Dsred) embryo injected with tmem33 morpholino displaying circulating erythrocytes within the DA. Elapsed time from the start point of imaging is in seconds, anterior is left, lateral view.

**File Name:** Supplementary Movie 3

**Description:** Circulating erythrocytes in global tmem33 crispant at 48hpf Time lapse- recording of 3D rendered lightsheet images of Tg(fli1a:EGFP;gata1a:Dsred) embryo injected with tmem33 sgRNAs and dCas9 mRNA and displaying circulating erythrocytes within the DA. Anterior is left, lateral view.

**File Name:** Supplementary Movie 4

**Description:** Ca2+ oscillations in migrating SeAs in control morphants at 28hpf Time-lapse recording of 3D-rendered light sheet images of Tg(fli1a:gal4ff;uas-GCaMP7a) embryo injected with control morpholino. Fluorescence intensity map is shown. Elapsed time from the start point of imaging is in seconds, anterior is left, lateral view.

**File Name:** Supplementary Movie 5

**Description:** Ca2+ oscillations in migrating SeAs in tmem33 morphants at 28hpf Time-lapse recording of 3D-rendered light sheet images of Tg(fli1a:gal4ff;uas-GCaMP7a) embryo injected with tmem33 morpholinos. Fluorescence intensity map is shown. Elapsed time from the start point of imaging is in seconds, anterior is left, lateral view.

**File Name:** Supplementary Movie 6

**Description:** Ca2+ oscillations in migrating SeAs in uninjected control at 26hpf Time-lapse recording of 3D-rendered light sheet images of Tg(fli1a:gal4ff;uas-GCaMP7a) embryo. Fluorescence intensity map is shown. Elapsed time from the start point of imaging is in seconds, anterior is left, lateral view.

**File Name:** Supplementary Movie 7

**Description:** Ca2+ oscillations in migrating SeAs at 26hpf following vegfa165 overexpression Time-lapse recording of 3D-rendered light sheet images of Tg(fli1a:gal4ff;uas-GCaMP7a) embryo injected with vegfa165 mRNA. Fluorescence intensity map is shown. Elapsed time from the start point of imaging is in seconds, anterior is left, lateral view.

**File Name:** Supplementary Movie 8

**Description:** Ca2+ oscillations in migrating SeAs at 26hpf in tmem33 crispant Time-lapse recording of 3D-rendered light sheet images of Tg(fli1a:gal4ff;uas-GCaMP7a) embryo injected with tmem33 sgRNAs and dCas9 mRNA. Fluorescence intensity map is shown. Elapsed time from the start point of imaging is in seconds, anterior is left, lateral view.

**File Name:** Supplementary Movie 9

**Description:** Ca2+ oscillations in migrating SeAs at 26hpf in tmem33 crispant following vegfa165 overexpression Time-lapse recording of 3D-rendered light sheet images of Tg(fli1a:gal4ff;uas-GCaMP7a) embryo injected with vegfa165 mRNA, tmem33 sgRNAs and dCas9 mRNA . Fluorescence intensity map is shown. Elapsed time from the start point of imaging is in seconds, anterior is left, lateral view.

**File Name:** Supplementary Movie 10

**Description:** Migrating SeAs between 24-30hpf. Time-lapse recording of 3D-rendered light sheet images of Tg(kdrl:HRAS-mCherry-CAAX; flk1:EGFP-NLS) embryo. Elapsed time from the start point of imaging is in hours, anterior is left, lateral view.

**File Name:** Supplementary Movie 11

**Description:** Migrating SeAs between 24-30hpf during SKF96365 treatment. Time-lapse recording of 3D-rendered light sheet images of Tg(kdrl:HRAS-mCherry-CAAX; flk1:EGFP-NLS) embryo treated with 50μM SKF96365 from 21-30hpf. Elapsed time from the start point of imaging is in hours, anterior is left, lateral view.
