# Supplementary material for: tmem33 is essential for VEGF-mediated endothelial calcium oscillations and angiogenesis
Source: Nat Commun. 2019 Feb 13;10:732. doi: 10.1038/s41467-019-08590-7 (PMC6374405; doi:10.1038/s41467-019-08590-7)
Supplement: Supplementary file 15 — Reporting Summary [file 41467_2019_8590_MOESM15_ESM.pdf]

nature research

Corresponding author(s): Robert N. Wilkinson

Last updated by author(s): 18/12/2018

Reporting Summary

Nature Research wishes to improve the reproducibility of the work that we publish. This form provides structure for consistency and transparency in reporting. For further information on Nature Research policies, see Authors & Referees and the Editorial Policy Checklist.

Statistics

For all statistical analyses, confirm that the following items are present in the figure legend, table legend, main text, or Methods section.

n/a	Confirmed	
☐	☒	The exact sample size (*n*) for each experimental group/condition, given as a discrete number and unit of measurement
☐	☒	A statement on whether measurements were taken from distinct samples or whether the same sample was measured repeatedly
☐	☒	The statistical test(s) used AND whether they are one- or two-sided<br>*Only common tests should be described solely by name; describe more complex techniques in the Methods section.*
☐	☒	A description of all covariates tested
☐	☒	A description of any assumptions or corrections, such as tests of normality and adjustment for multiple comparisons
☐	☒	A full description of the statistical parameters including central tendency (e.g. means) or other basic estimates (e.g. regression coefficient) AND variation (e.g. standard deviation) or associated estimates of uncertainty (e.g. confidence intervals)
☐	☒	For null hypothesis testing, the test statistic (e.g. *F*, *t*, *r*) with confidence intervals, effect sizes, degrees of freedom and *P* value noted<br>*Give P values as exact values whenever suitable.*
☒	☐	For Bayesian analysis, information on the choice of priors and Markov chain Monte Carlo settings
☒	☐	For hierarchical and complex designs, identification of the appropriate level for tests and full reporting of outcomes
☒	☐	Estimates of effect sizes (e.g. Cohen's *d*, Pearson's *r*), indicating how they were calculated

Our web collection on statistics for biologists contains articles on many of the points above.

Software and code

Policy information about availability of computer code

Data collection	Images were collected using Zen Black for lightsheet 2014, Zen 2 (blue edition v1), Volocity V5.3.2, Leica LCS
Data analysis	Images were analysed using multiple different versions of FIJI throughout the course of the study, statistical analysis was performed using GraphPad Prism versions 5-7 and GPower version 3. Dorsal aorta blood flow velocimetry was quantified using MatLab version 9 using a custom algorithm freely available upon request and described in the methods.

For manuscripts utilizing custom algorithms or software that are central to the research but not yet described in published literature, software must be made available to editors/reviewers. We strongly encourage code deposition in a community repository (e.g. GitHub). See the Nature Research guidelines for submitting code & software for further information.

Data

Policy information about availability of data

All manuscripts must include a data availability statement. This statement should provide the following information, where applicable:

- Accession codes, unique identifiers, or web links for publicly available datasets
- A list of figures that have associated raw data
- A description of any restrictions on data availability

The datasets generated during and/or analysed during the current study are available from the corresponding author on reasonable request. The source data underlying Figs 1L-M, 2B, D, E, H, I, 3G, J, M, P 4E, H, I, 5A-C, 6C, F, G, H, U, and 7C, F, G, J, M, P and Supplementary Figs 2E, 3D, 4D, E, 5B, D, F, H, I, J, 6C, F, 7E, H, L, O, 8C, E, G, H, K, L, M, 9J, K 10K, L, 12C, F, 13C, F, G, J, M and 14E-H are provided as a Source Data file

Field-specific reporting

Please select the one below that is the best fit for your research. If you are not sure, read the appropriate sections before making your selection.

☒ Life sciences ☐ Behavioural & social sciences ☐ Ecological, evolutionary & environmental sciences

For a reference copy of the document with all sections, see nature.com/documents/nr-reporting-summary-flat.pdf

Life sciences study design

All studies must disclose on these points even when the disclosure is negative.

Sample size	Sample sizes were determined using a priori or post hoc power calculations
Data exclusions	No data were excluded from the analyses
Replication	All attempts at replication were successful
Randomization	Samples were randomly allocated into experimental groups
Blinding	Wherever possible analysis was performed blinded. Where this was not possible for example, when dominant markers were used to select a particular genotype, embryos were selected at random

Reporting for specific materials, systems and methods

We require information from authors about some types of materials, experimental systems and methods used in many studies. Here, indicate whether each material, system or method listed is relevant to your study. If you are not sure if a list item applies to your research, read the appropriate section before selecting a response.

Materials & experimental systems

n/a	Involved in the study	
☐	☒	Antibodies
☒	☐	Eukaryotic cell lines
☒	☐	Palaeontology
☐	☒	Animals and other organisms
☒	☐	Human research participants
☒	☐	Clinical data

Methods

n/a	Involved in the study	
☒	☐	ChIP-seq
☒	☐	Flow cytometry
☒	☐	MRI-based neuroimaging

Antibodies

Antibodies used	anti-CRISPR/Cas9 (7A9-3A3) (Novus Biologicals, NBP2-36440, 1:100), chicken anti-GFP (Abcam, ab13970, 1:500) primary antibodies and goat anti-mouse IgG (H&L) Alexa Fluor® 647 (Thermo Fisher A21235, 1:1000), goat anti-chicken IgY (H&L) Alexa Fluor® 488 (Thermo Fisher A11039, 1:1000) secondary antibodies, Phospho-p44/42 Erk1.2 (Thr202.Tyr204) Rabbit mAb (#4370, cell signal, 1:250)
Validation	Phospho-p44/42 Erk1.2 (Thr202.Tyr204) Rabbit mAb (#4370, cell signal, 1:250) Antibody validated as specific to ERK using using ERK inhibitor SL327<br>anti-CRISPR/Cas9 (7A9-3A3) (Novus Biologicals, NBP2-36440, 1:100) Antibody validated as specific using parallel non-dCas9 expressing embryos as negative control<br>chicken anti-GFP (Abcam, ab13970, 1:500) antibody validated as specific using parallel non GFP positive embryos as negative control.

Animals and other organisms

Policy information about studies involving animals; ARRIVE guidelines recommended for reporting animal research

Laboratory animals	The study employed zebrafish (danio rerio) embryos produced from matings between male and female wild type strains including AB, TL and LWT and transgenic strains generated in these backgrounds.
Wild animals	the study did not involve wild animals
Field-collected samples	The study did not contain samples collected from the field

Ethics oversight	All animal experiments were performed in accordance with UK Home office regulations under project licence 40/3708 and 70/8588

Note that full information on the approval of the study protocol must also be provided in the manuscript.
